# Supplementary material for: Domesticated cannabinoid synthases amid a wild mosaic cannabis pangenome
Source: Nature. 2025 May 28;643(8073):1001–10. doi: 10.1038/s41586-025-09065-0 (PMC12286863; doi:10.1038/s41586-025-09065-0)
Supplement: Supplementary file 1 — Supplementary Figures, Supplementary Tables, Supplementary Notes 1–3 and references [file 41586_2025_9065_MOESM1_ESM.pdf]

nature portfolio

https://doi.org/10.1038/s41586-025-09065-0

Supplementary information

Domesticated cannabinoid synthases amid a wild mosaic cannabis pangenome

In the format provided by the authors and unedited

Cannabis Pangenome Supplementary Materials

Title:
Domesticated cannabinoid synthases amid a wild mosaic cannabis pangenome

Short Title:
The Cannabis Pangenome

Authors:
Ryan C. Lynch[1]*, Lillian K. Padgitt-Cobb[1]*, Andrea R. Garfinkel[2], Brian J. Knaus[3], Nolan T. Hartwick[1], Nicholas Allsing[1], Anthony Aylward[1], Philip C. Bentz[4], Sarah B. Carey[4], Allen Mamerto[1], Justine K. Kitony[1], Kelly Colt[1], Emily R. Murray[1], Tiffany Duong[1], Heidi I. Chen[1], Aaron Trippe[2], Alex Harkess[4], Seth Crawford[2], Kelly Vining[3], Todd P. Michael[1,5,6]

Affiliations:
[1]The Plant Molecular and Cellular Biology Laboratory, The Salk Institute for Biological Studies, La Jolla, CA 92037, USA
[2]Oregon CBD, Independence, OR 97351, USA
[3]Department of Horticulture, Oregon State University, Corvallis, OR 97331, USA
[4]HudsonAlpha Institute for Biotechnology, Huntsville, AL 35806, USA
[5]Department of Cell and Developmental Biology, School of Biological Sciences, and Center for Marine Biotechnology and Biomedicine, University of California, San Diego, La Jolla, CA, 92093, USA
[6]Department of Science and Conservation, San Diego Botanical Garden, Encinitas, CA, 92024, USA

Correspondence:
Todd P. Michael, tmichael@salk.edu, toddpmichael@gmail.com
Ryan C. Lynch rlynch@colorado.edu
Lillian K. Padgitt-Cobb lilliankpc@gmail.com

Supplementary Figures..3
Supplementary Tables..33
Supplementary Note 1: EH23a and F2 mapping population...49
Supplementary Note 2: Terpene and disease resistance genes.. 50
Supplementary Note 3: WGD analysis reveals cannabis has the eudicot hexaploidization.. 50
Supplementary References.. 52

Supplementary Figures

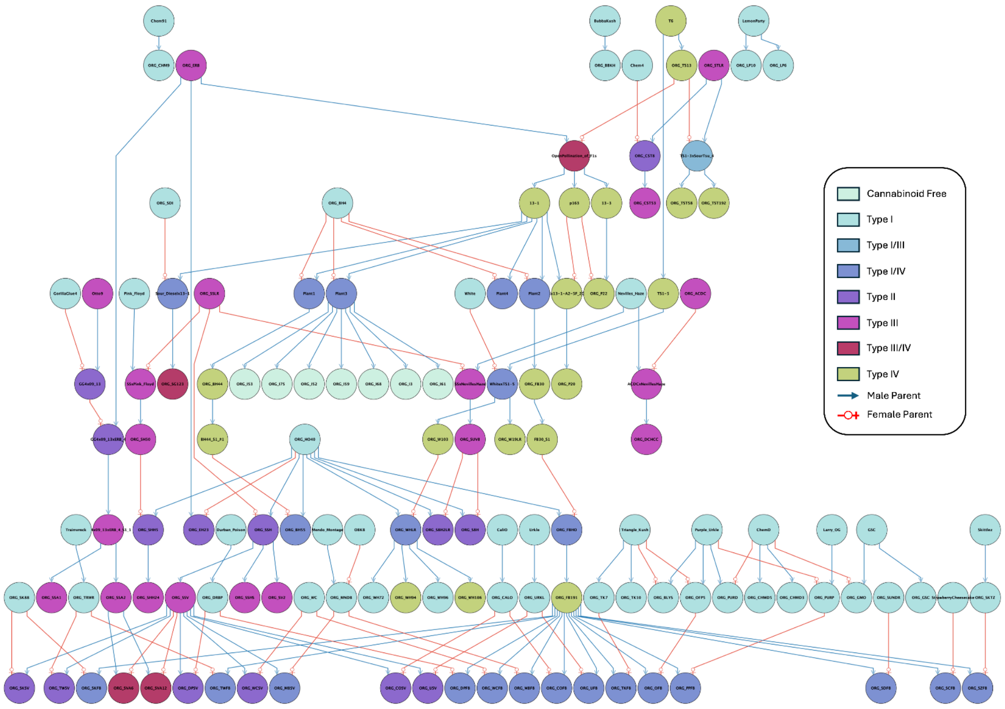

Supplementary Figure 1. The Pedigree of the OCBD cultivars sequenced for the cannabis pangenome. A high proportion of the pangenome is based on the Oregon CBD (OCBD) breeding program including a specific strategy to produce high varin lines**.** Pedigree of the OCBD breeding program to generate high varin CBGA plants. HO40, which is a high varin plant, was crossed with the high CBGA plant FB30_S1 (100:1 CBGA:THCA, DN dominant) and selfed. Then, a high varin plant was selected (FB191) for crossing to type I plants for high CBGV production. **FigShare link:** https://doi.org/10.6084/m9.figshare.27981758.v1

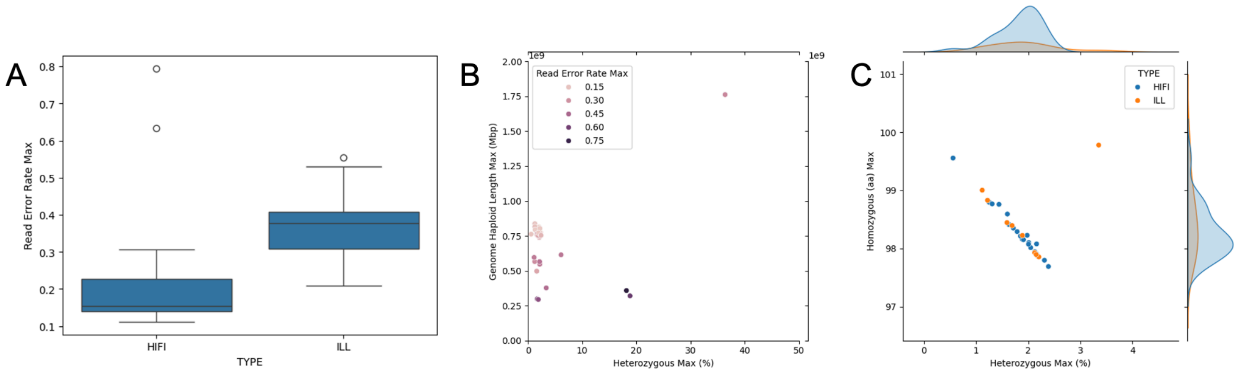

Supplementary Figure 2. Heterozygosity at the SNP level in the cannabis pangenome. We selected a subset of PacBio HiFi and Illumina short reads to estimate heterozygosity. A) The HiFi reads had a slightly lower error rate compared to the Illumina reads. B) Haploid genome length (max prediction) versus heterozygosity (max prediction). C) . Homozygous (“aa” max prediction) versus heterozygosity (max prediction).

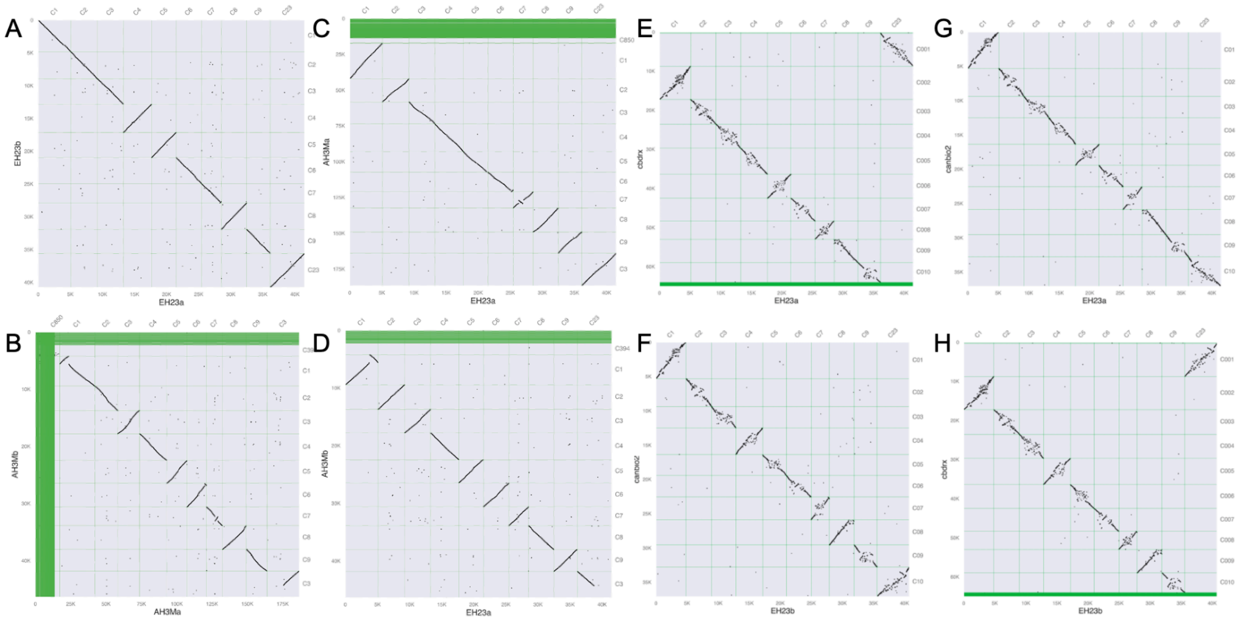

Supplementary Figure 3. Protein based dotplots comparing new cannabis pangenome assemblies to CBDRx. All dotplots were generated with MCscan python version using synteny between the predicted protein coding genes**.** A) EH23a vs. EH23b; B) AH3Ma vs. AH3Mb; C) AH3Ma vs. EH23a; D) AH3Mb vs. EH23a; E) EH23a vs. CBDRx; F) EH23b vs. CBDRx; G) EH23a vs. Cannbio; H) EH23b vs. Cannbio.

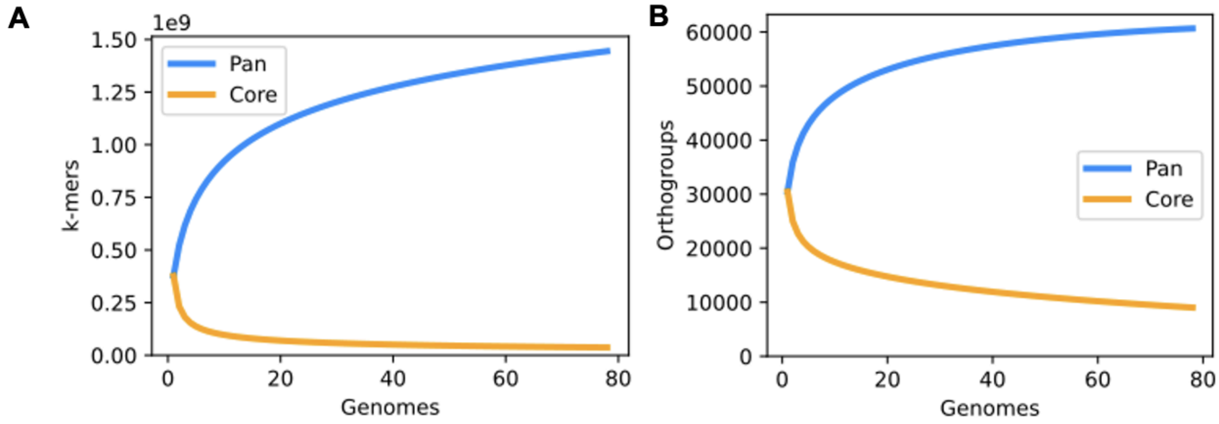

Supplementary Figure 4. Collector's curves (pangenome curves) for the 78 haplotype-resolved, chromosome-scale assemblies. A) The collector's curve is based on PanKmer (31 bp K-mer). B) The collectors curve is based on orthofinder gene families.

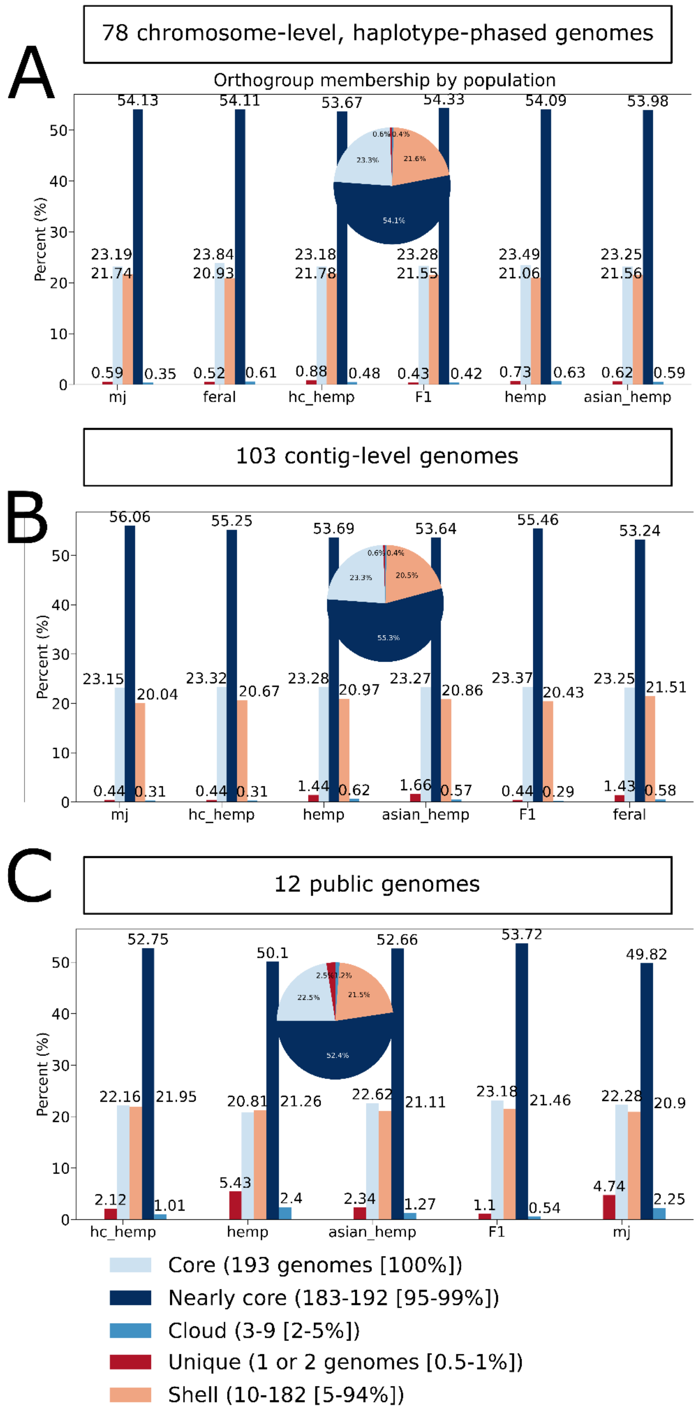

Supplementary Fig. 5. Core and dispensable genes in scaffolded genomes, contig-level genomes, and public genomes. Core and "nearly-core" orthogroups make up the majority of the pangenome, and unique ("private") genes are more variable. The 78 genomes are organized by haplotype-resolved pairs. The groupings of 103 genomes and 12 public genomes are each alphabetically arranged. The inset pie chart provides the aggregate percentages for each grouping. A) Bar chart showing population assignments and gene groupings for 78 chromosome-level, haplotype-resolved genomes; B) Bar chart showing population assignments

and gene groupings for 103 genomes; and C) Bar chart showing population assignments and gene groupings for 12 public genomes.

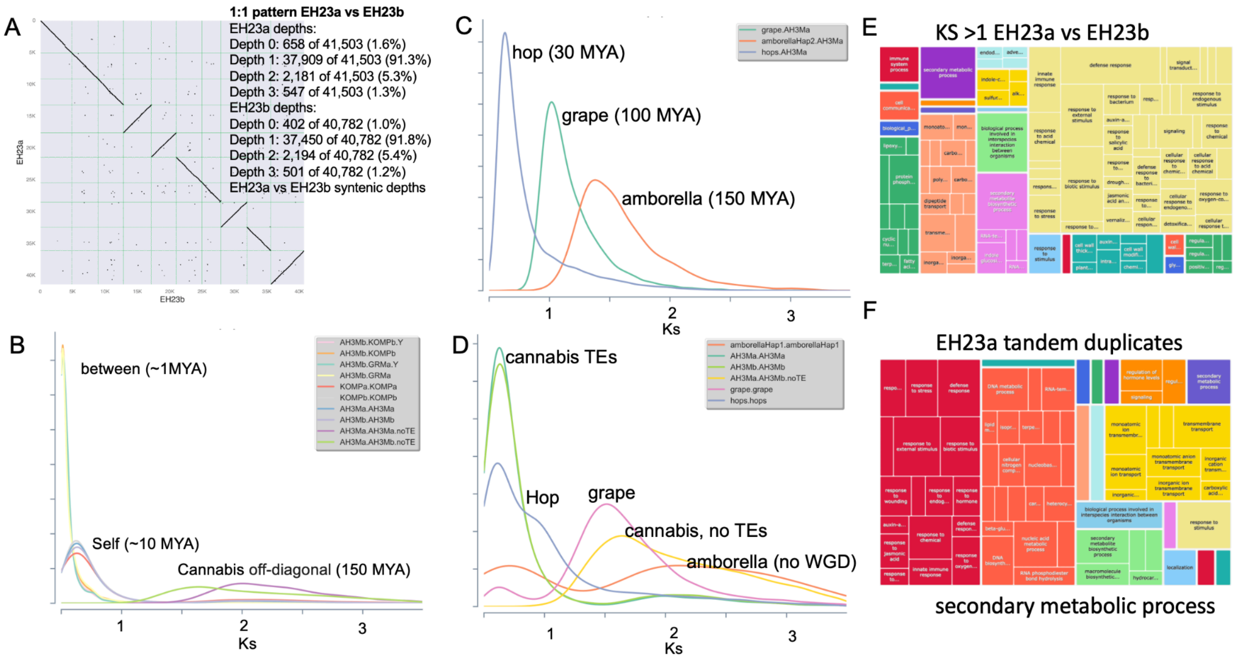

Supplementary Fig. 6. Whole genome duplication (WGD) history of cannabis is consistent with the lambda WGT ~100 mya and a recent burst of TEs and TDs. A) Protein-based dotplot comparing EH23a (HO40) with EH23b (ERB). The inset provides the syntenic pattern numbers showing that both genomes have few unique genes (~1%) not found in the other genome. 91% of genes are found in 1:1 syntenic relationship between the two haplotypes consistent with very few genes retained after whole genome duplication (WGD). B) The Ka/Ks (non-synonymous/synonymous substitution rate ratio) was estimated across several genomes in the pangenome and Ks was used to estimate the divergence time between genomes. The Ks between cannabis haplotypes shows a peak around 1 million years ago (MYA). In contrast, self-self Ks suggests a peak around 10 MYA. When TE-associated genes are removed, the off-diagonal Ks peak moves in line with the lambda whole genome triplication (WGT) found in many eudicots. C) AH3Ma haplotype was compared to amborella, which is sister to the eudicots and is typically used as a baseline for no WGD, and grape, which only has the lambda WGT. These Ks plots show the divergence between the two species is consistent with the Ks peaks. Cannabis diverged from hop ~30 MYA, from grape ~100 MYA around the lambda WGD, and from amborella 150 MYA. D) The timing of the last WGD was refined by looking at the self-self Ks values per species. E) The genes with a Ks > 1 are thought to be under positive selection. Gene ontology (GO) enrichment was performed for the genes with a Ks > 1 and the resulting GO categories were plotted using the tree-map function in Revigo. F) The cannabis genome has a large number of tandemly duplicated (TD) genes. Gene ontology (GO) enrichment was performed for the TD genes and the resulting GO categories were plotted using the tree-map function in Revigo.

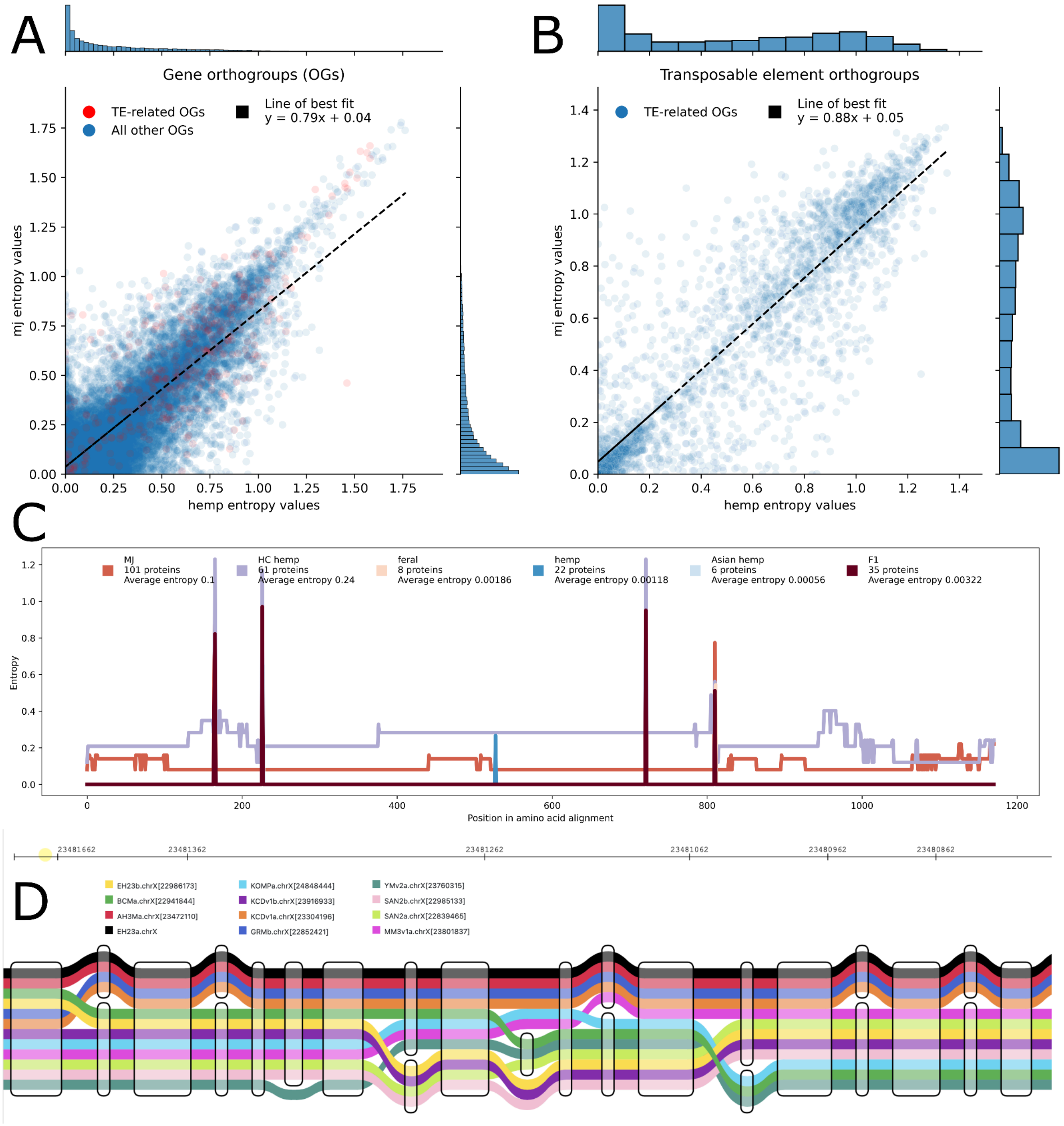

Supplementary Fig. 7. Entropy calculation for orthogroup protein sequences reveals patterns of selection across the pangenome. A) Average entropy values calculated from orthogroup multiple sequence alignments for each of the population assignments. This analysis includes the full pangenome. The red scatter dots correspond to orthogroups containing genes with similarity to TEs. MJ proteins show slightly higher entropy than hemp near zero. B) Average entropy values calculated from orthogroup multiple sequence alignments for each of the population assignments. C) Entropy values plotted for each position in the multiple sequence alignment for *GIGANTEA* on the X chromosome. D) Pangenome tube graph for a segment of the X chromosome *GIGANTEA*, including 12 genomes (SAN2a/b, KOMPa/b, KCDv1a/b, GRMa/b, BCMa/b, AH3Ma/b). This tube graph is generated from a reference-free pangenome graph of these genomes, reflective of different population types.

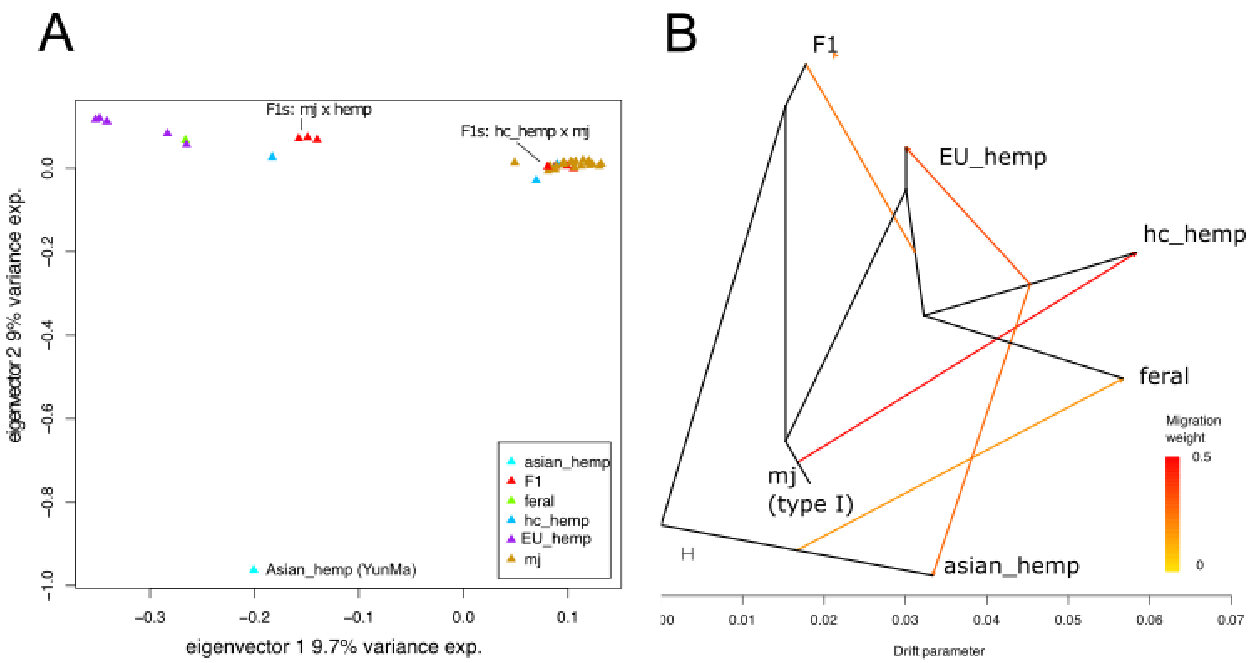

Supplementary Fig. 8. Cannabis pangenome analysis of the 78 haplotype-resolved, chromosome-scale assemblies for breeding history. A) Principal component analysis (PCA) of 494,603 high confidence phased SNPs from 78 haplotype-resolved, chromosome-scale assemblies (39 samples). Population assignments are based on pedigree and provenance, as well as PCA. This analysis appears to reflect differences between European hemp, Asian hemp and drug-type samples, but shows little differentiation among drug-type samples; therefore, we assigned all drug-type samples as either high cannabinoid (hc) hemp or marijuana (MJ) for downstream SNP-based analyses. Samples that are known breeding program hybrids between these major groups were classified as F1s. B) TreeMix SNP based phylogeny shows likely relationships between major populations including 5 hybridization events, based on SNPs from 78 haplotype-resolved, chromosome-scale assemblies.

Supplementary Fig. 9. Global cannabis tree of life. Hierarchical clustering (average) of 31-mer based similarities among all 193 pangenome assemblies and Illumina short-read libraries from Ren et al. 2021 [4]. https://doi.org/10.6084/m9.figshare.27883008.v1

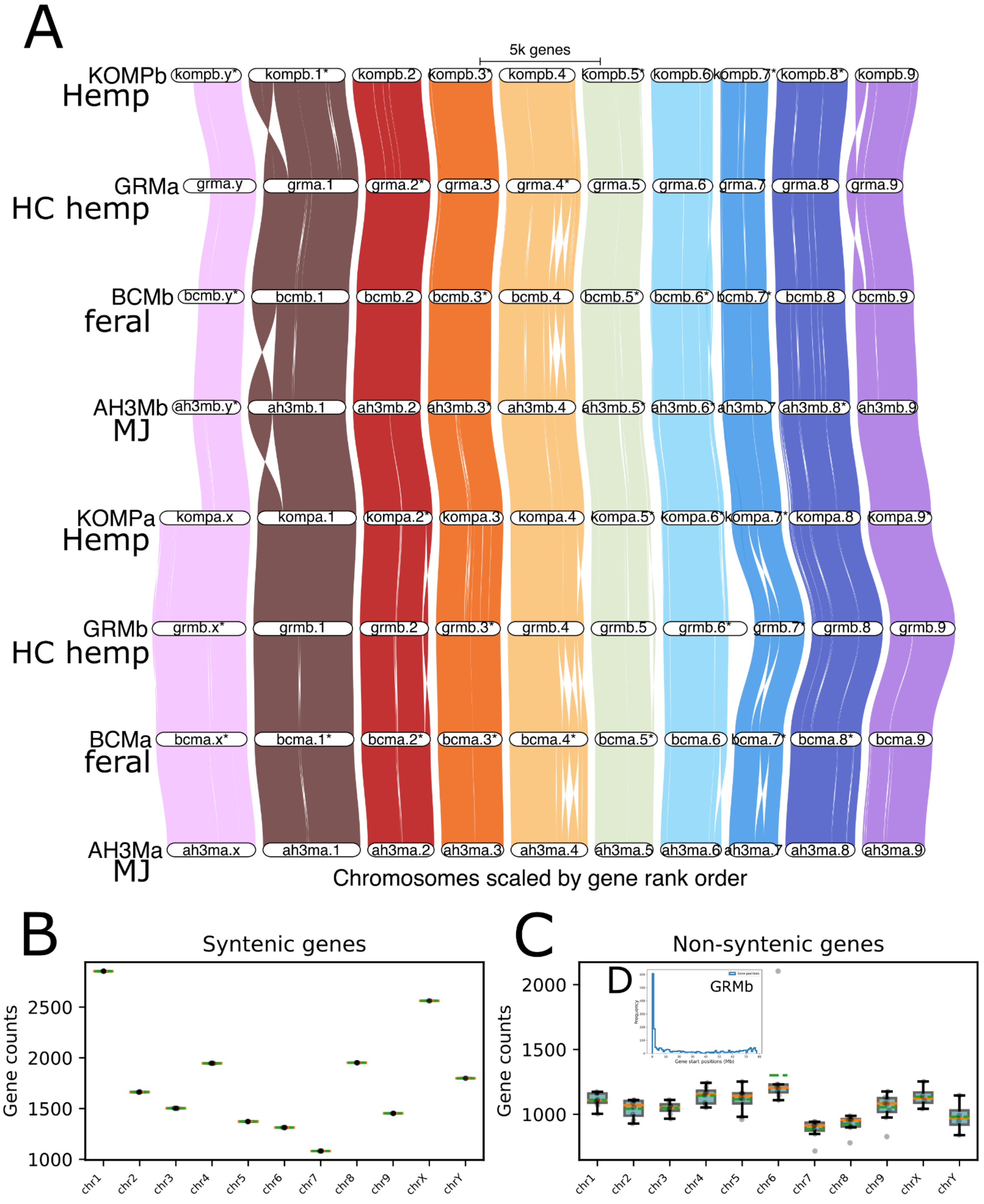

Supplementary Fig. 10. Visualization of synteny in eight haplotype-resolved, chromosome-scale assemblies (from four samples) with X and Y chromosomes, as well as nine autosomes (AH3Ma/b, BCMa/b, GRMa/b, KOMPa/b), belonging to different populations (hemp, high cannabinoid [HC] hemp, MJ, feral). A) Syntenic genes in eight genomes drawn with genespace. Chromosomes are not drawn to scale based on physical positions; positions on the x-axis are scaled by gene rank order. The pseudoautosomal region

(PAR) boundary is drawn between KOMPa chromosome X and AH3Mb chromosome Y. Chromosome 1 has a large inversion, and there are smaller inversions in other chromosomes. However, gene-based synteny is broadly consistent across genomes. B) Syntenic gene counts for the eight genomes. C) Non-syntenic gene counts for the eight genomes. D) Figure inset as part of panel C: GRMb is annotated as an outlier, with 2,104 non-syntenic genes at the beginning of chromosome 6, most of which lack similarity to a known gene, or have similarity to ribosomal or photosynthesis genes.

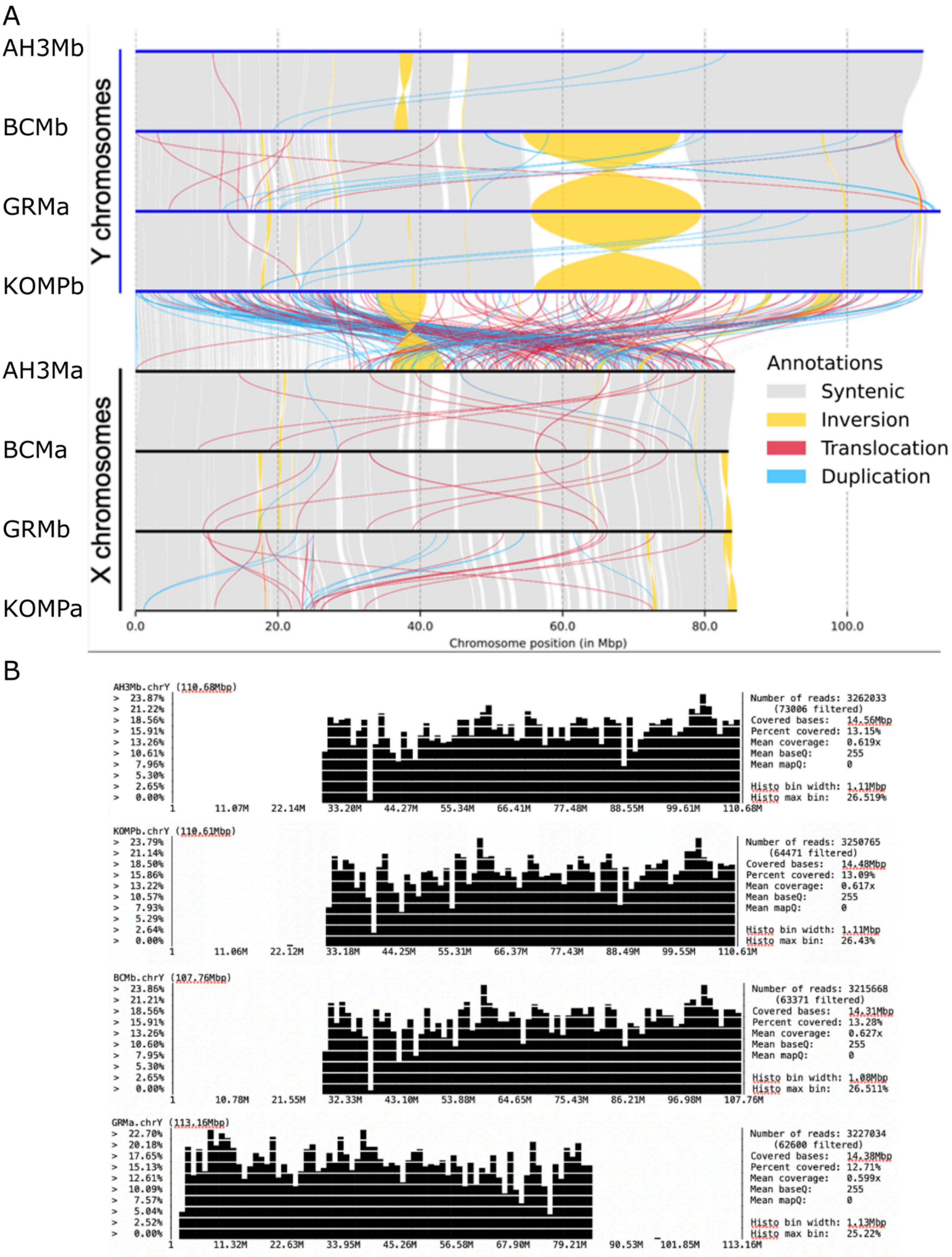

Supplementary Fig. 11. Structural variation and k-mer coverage in the sex chromosomes.
A) Y chromosome synteny and structural variation with select X chromosomes. Alignments

between X and Y chromosomes show syntenic regions (grey), inversions (yellow) translocations (red), and duplications (blue), and unalignable regions (white). We identified a large ~25 Mb inversion within the SDR of the Y chromosome (located ~24 Mb from the SDR-PAR boundary) in the hc hemp sample Golden Redwood (GRMa; type III), which is not predicted to suppress homologous recombination due to the lack of homology in the SDR to the X chromosome. Rather this large inversion may represent a neutral mutation perhaps caused by the elevated levels of TE activity or ectopic recombination in the SDR. This plot was generated from assembly-assembly alignments and synteny analysis with minimap2 [5] and syri [6], respectively, then plotted with plotsr [7]. B) Coverage plot of Y-specific k-mers in male genomes (AH3Mb, BCMb, GRMa, KOMPb). GRMa.chrY was not reverse complemented to match the orientation of the other genomes in this plot.

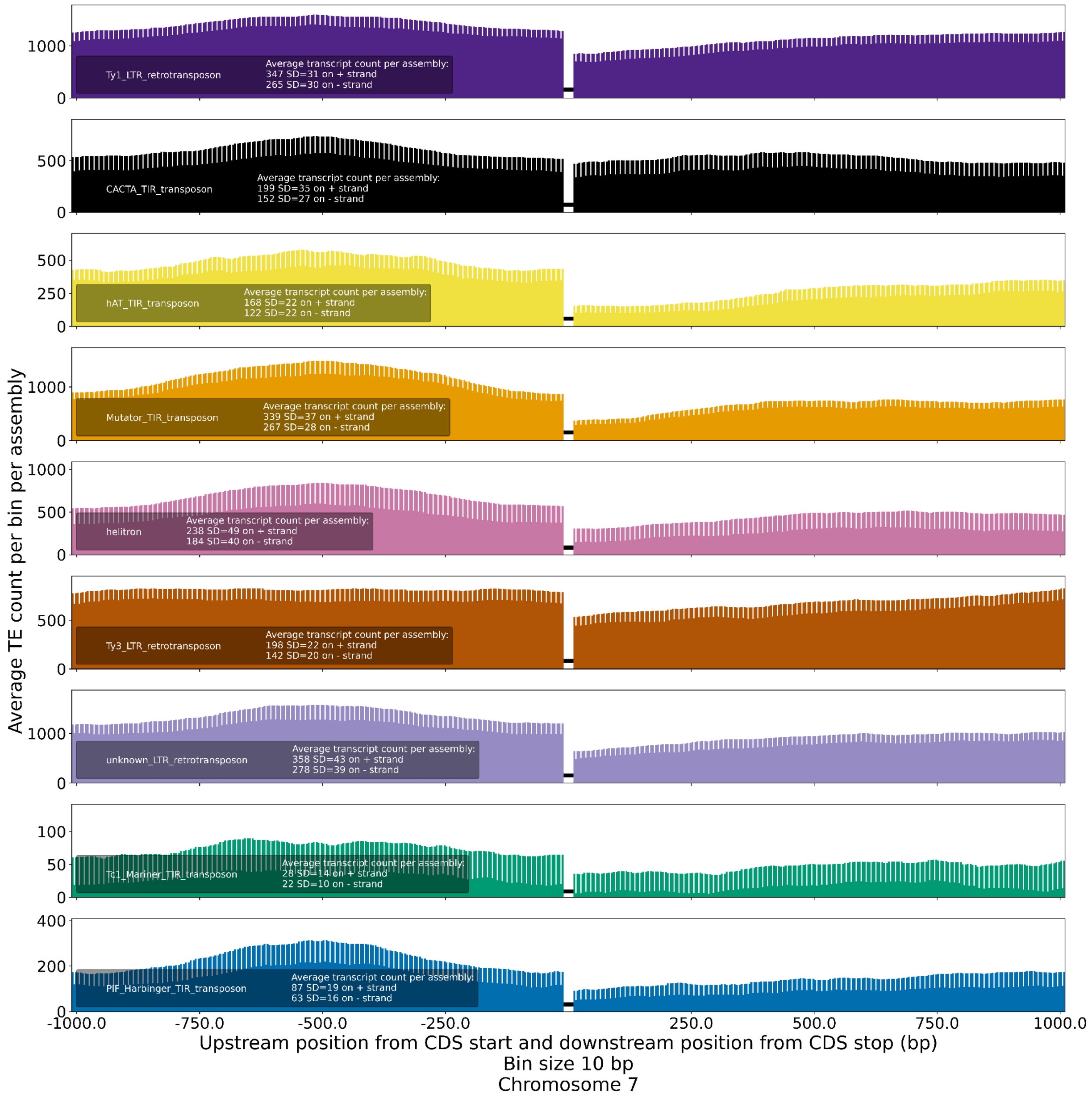

Supplementary Fig. 12. Position-specific density of transposable elements (TEs) upstream (1 kb) and downstream (1 kb) of genes in the cannabis pangenome (78 scaffolded genomes). Each panel corresponds to a different TE, with the histograms showing the average TE count across all scaffolded assemblies in the region upstream and downstream of genes, along with error bars. The horizontal line in the center of each of the panels denotes the gene body.

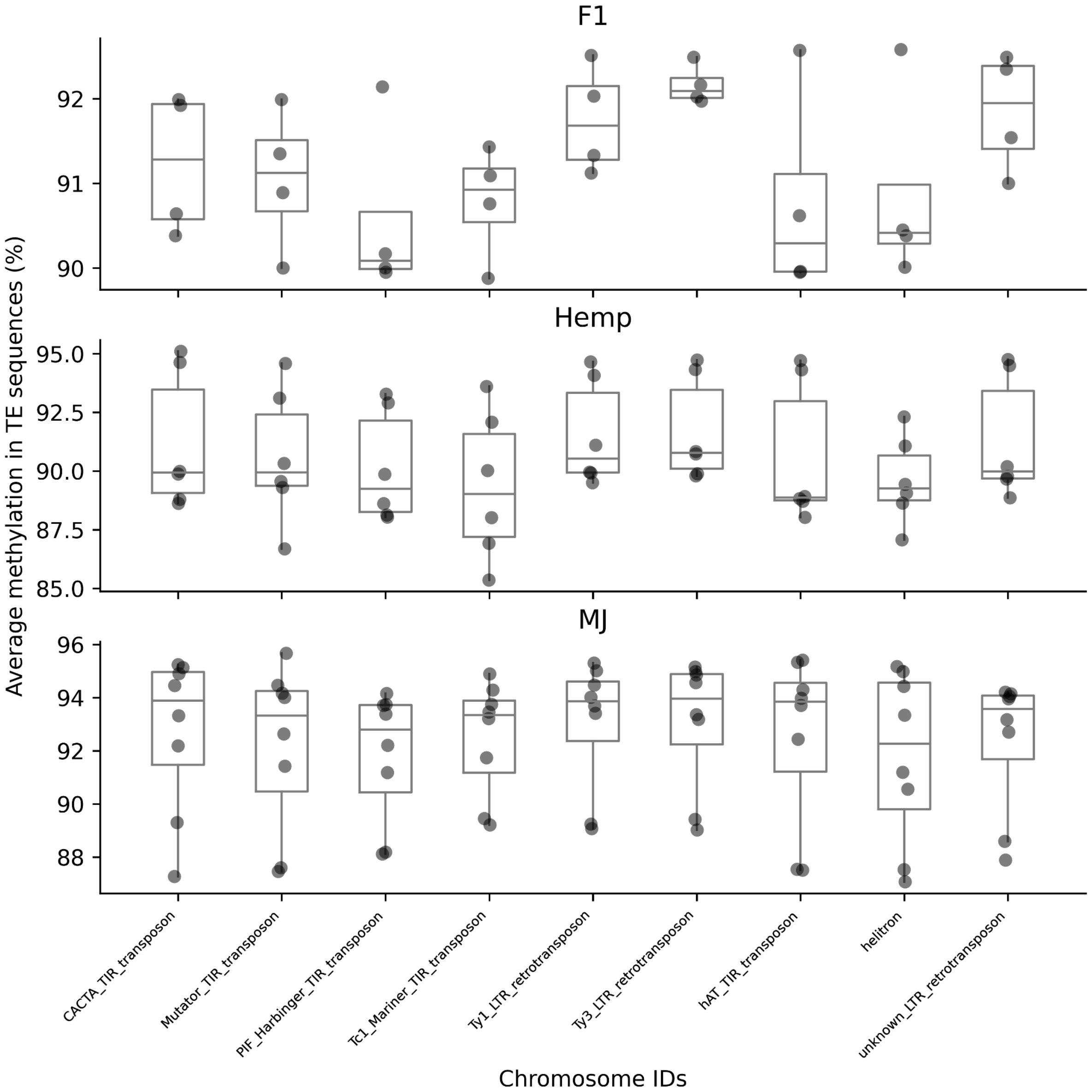

Supplementary Fig. 13. Average methylation (%) in TE sequences. Analysis applies to the subset of genomes with methylation data (see **Supplementary Table 12**). Each data point in the box plot corresponds to a specific assembly. For each genome, the average methylation value (%) along the length of a TE sequence was averaged for all TE sequences. MJ samples have a slightly higher average methylation value in the TEs than F1 samples.

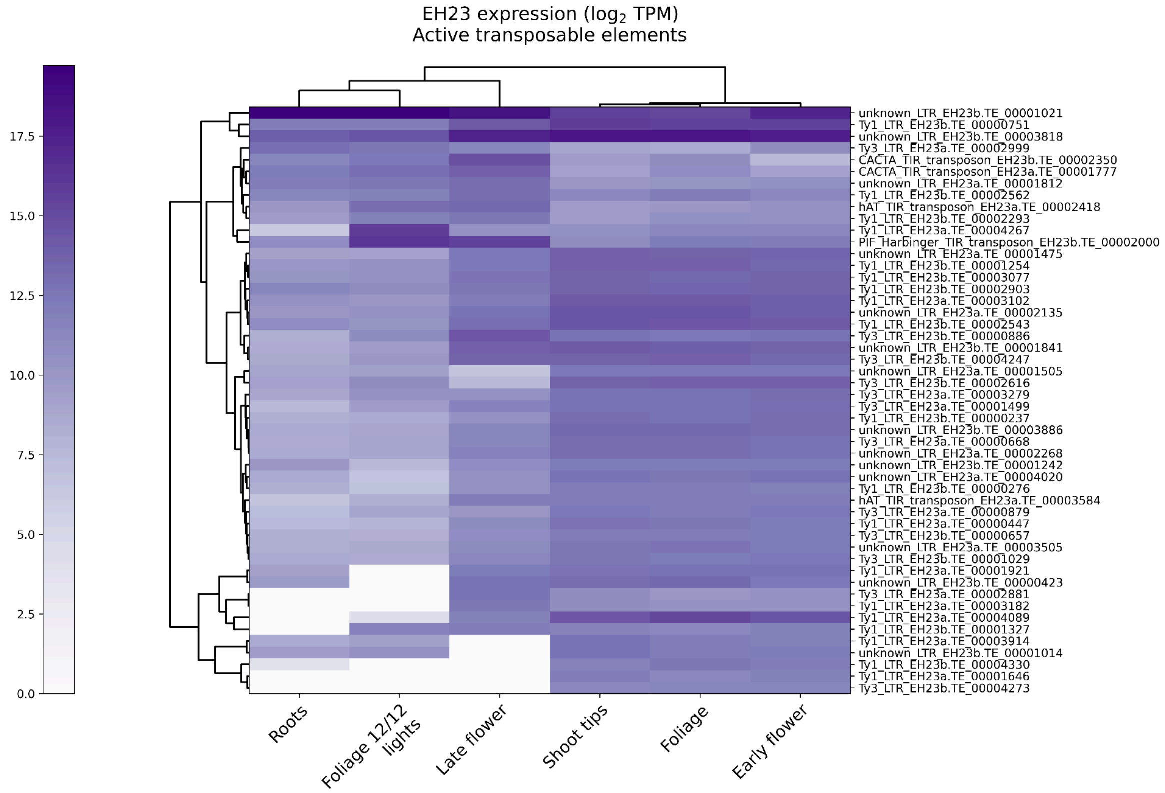

Supplementary Fig. 14. Heatmap showing top 50 expressed TEs in EH23. The majority of active TEs are LTRs, and putative active DNA TEs in EH23 include CACTA, hAT, and Harbinger. Active TEs show tissue-specific patterns, with late-stage flowers showing higher Harbinger activity and lacking activity that is present in shoot tips, foliage, and early-stage flowers.

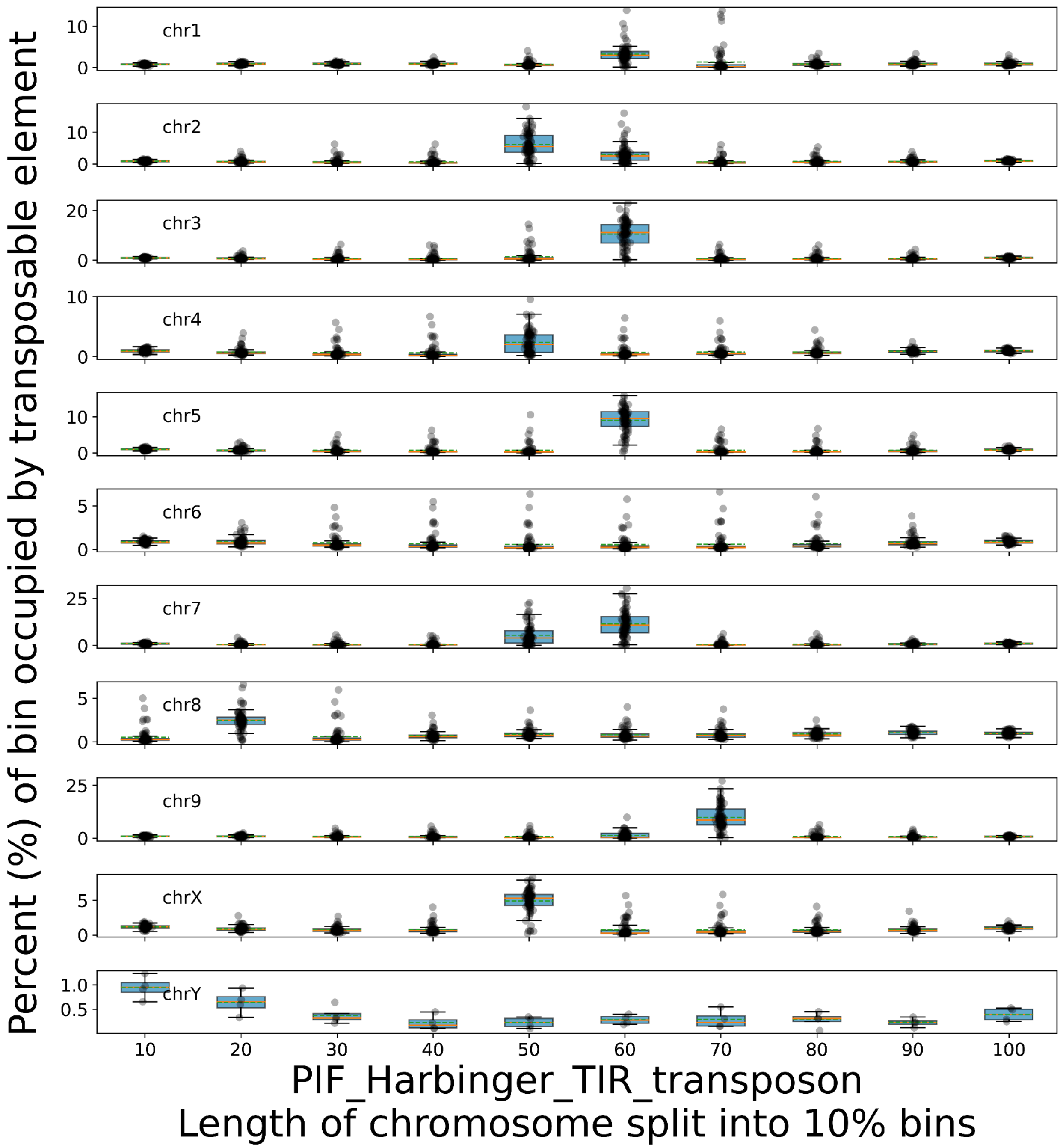

Supplementary Fig. 15. DNA transposable element Harbinger is a common feature of the cannabis centromeres and pericentromeres. Each of the chromosomes of the scaffolded assemblies is divided into sections/bins that each make up 10% of its sequence length, as a way to compare chromosomes with different lengths. Harbinger tends to occur in the center of chromosomes, corresponding to the putative centromere, except for chromosome 8, which is acrocentric.

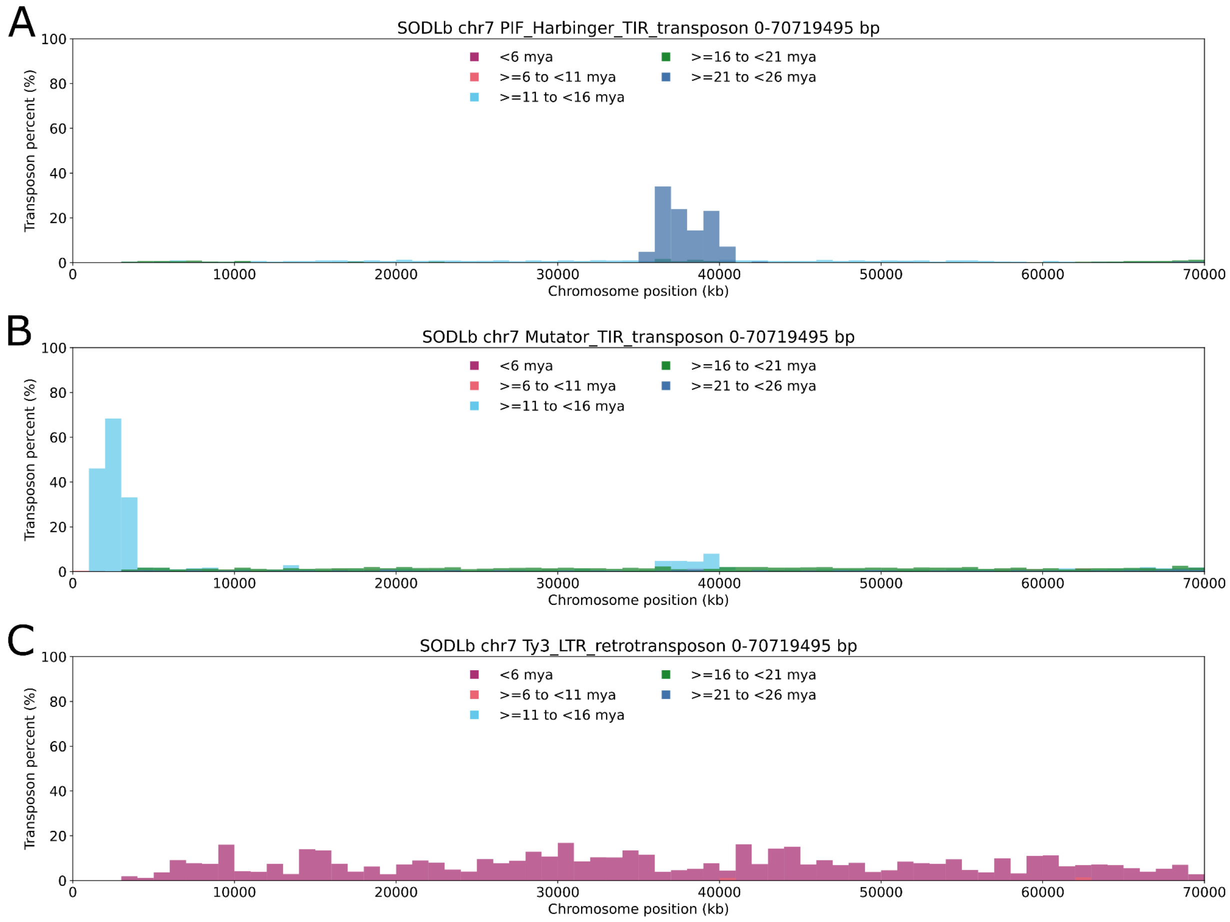

Supplementary Fig. 16. Distribution of TE divergence times across chromosome 7 of genome SODLb. DNA TEs have dynamic distributions that potentially reflect their role in shaping the genome over millions of years. DNA TE Harbinger occurs in the centromere region with a divergence date of ~20 million years, while Mutator is especially abundant in gene-rich regions, and is more recent than Harbinger. In contrast, Ty3-LTR-RTs proliferated recently across the chromosome, with lower abundance in gene-rich regions.

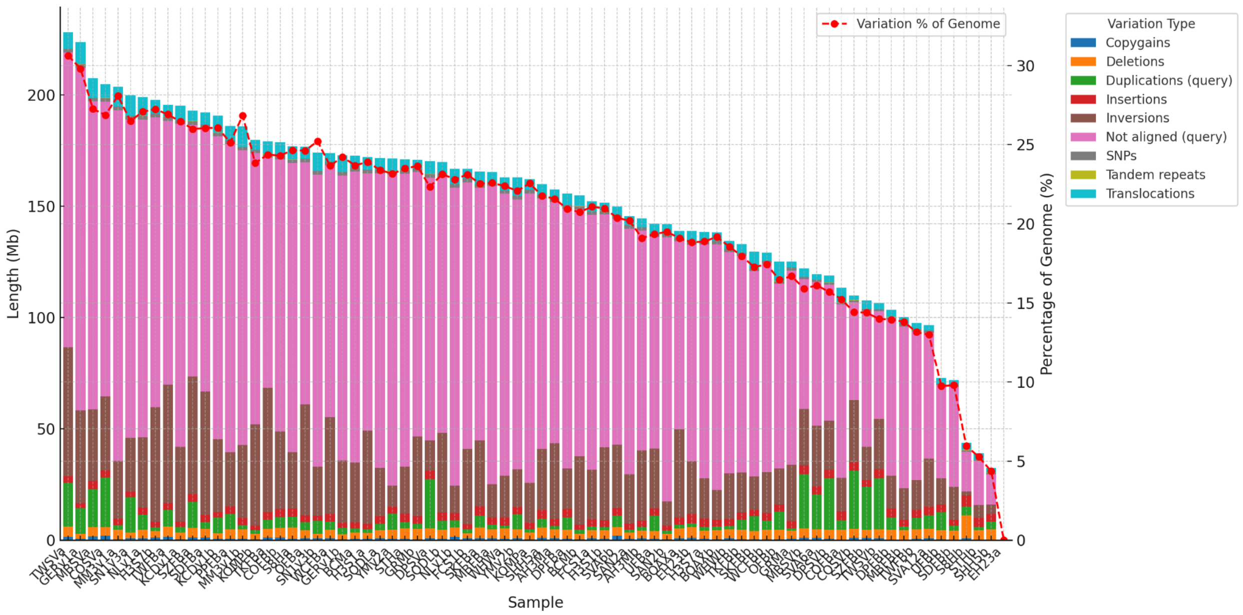

Supplementary Fig. 17. Total lengths of each variation type per sample. Bars and percent of total genome (red points) include unaligned regions of each query. Alignments based on pairwise alignments to EH23a haplotype.

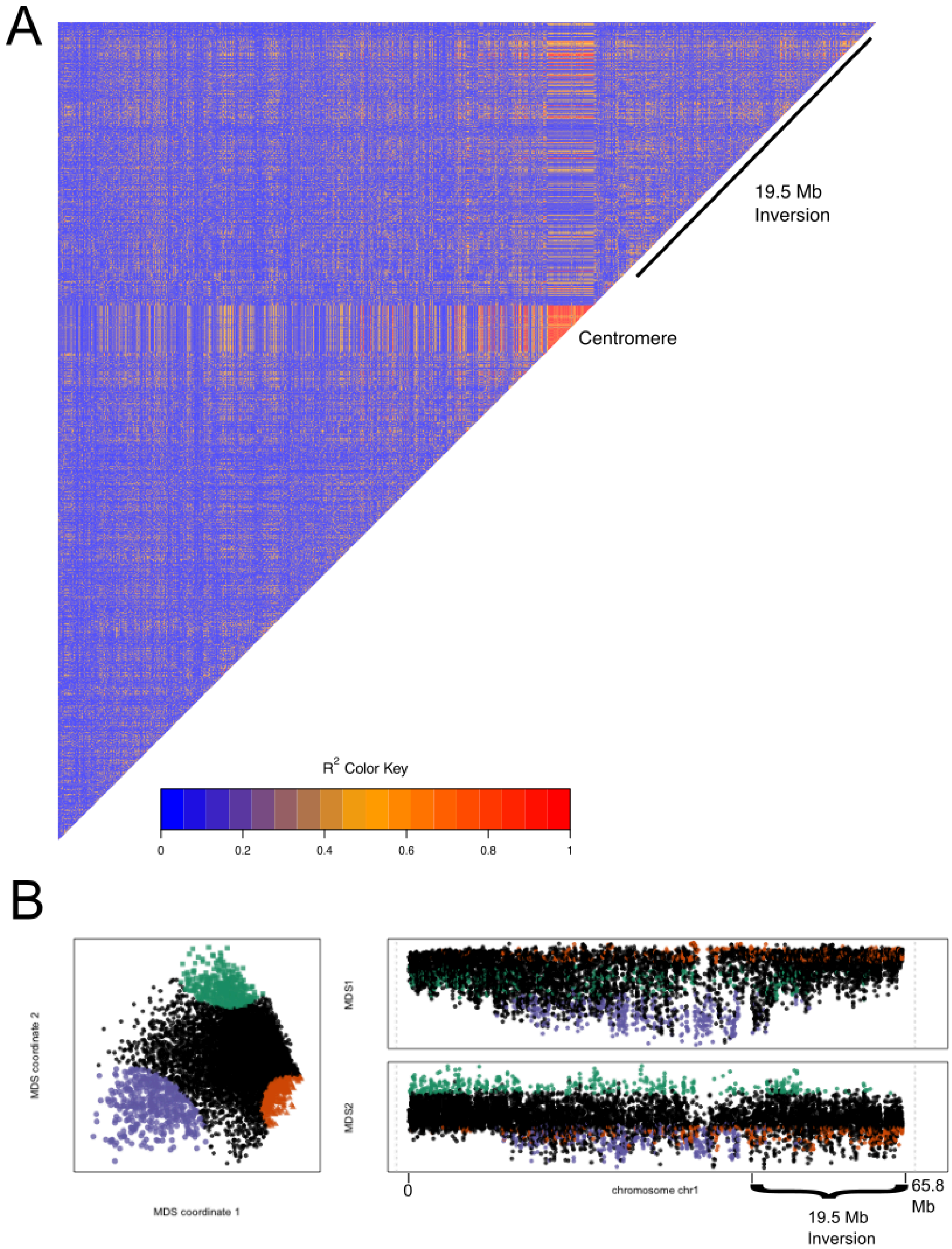
A
19.5 Mb
Inversion
Centromere
R^2 Color Key
0
0.2
0.4
0.6
0.8
1
B
MDS coordinate 2
MDS coordinate 1
MDS1
MDS2
0
chromosome chr1
65.8
Mb
19.5 Mb
Inversion

Supplementary Fig. 18. Linkage disequilibrium (LD) and local PCA identify a region on Chromosome 1 resulting in segregation distortion. A) Pairwise SNP R^2 heatmap of chromosome 1 showing elevated linkage disequilibrium in the centromeric region and within the large (~19.5 MB) inversion. B) Local PCA analysis highlighting a shift in SNP frequencies in the centromeric region and the interior inversion breakpoint. The two multidimensional scaling (MDS) axes of the PCA distances for 200 SNP genomic windows plotted along the EH23a chromosomes. Orange, purple and green points highlight three clusters of outlier windows.

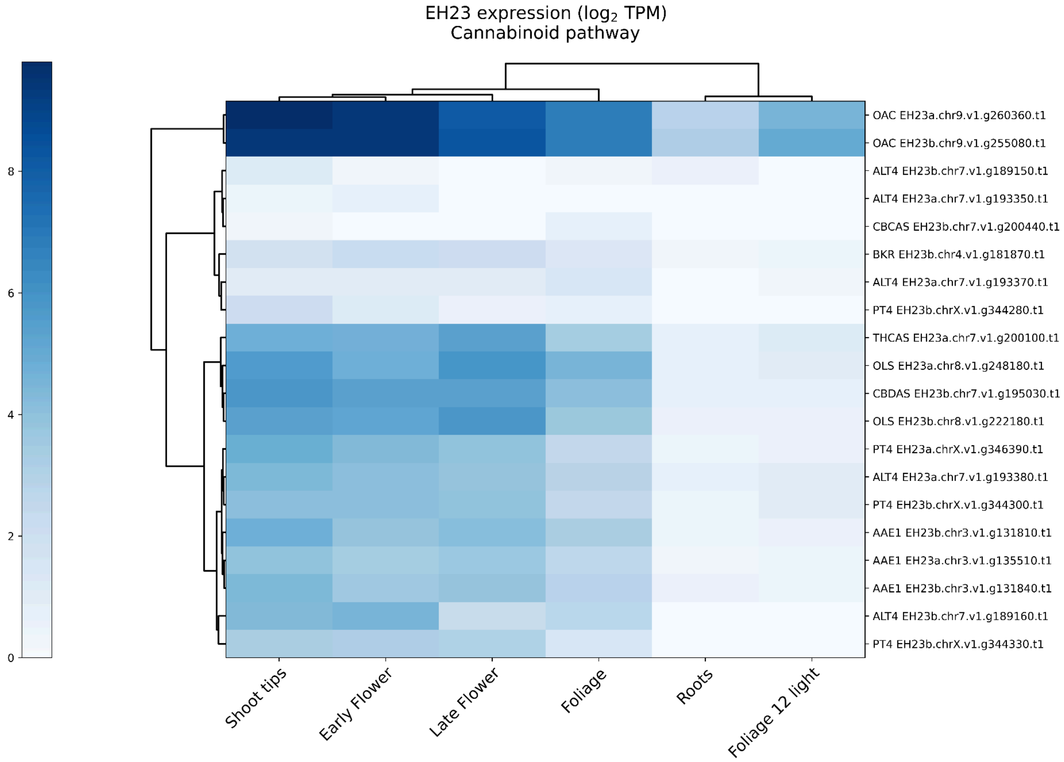

Supplementary Fig. 19. Expression of cannabinoid precursor pathway genes in EH23. Expression values for early and late-stage flowers are clustered together. *OAC (olivetolic acid cyclase*, UniProt ID I6WU39) is located on chromosome 9 and shows the highest expression. *ALT4* and *PT4* tend to occur together in the clustered expression heatmap, suggesting a pattern of co-expression and co-regulation. Pathway genes: *acyl-activating enzyme (AAE1), olivetolic acid synthase (OLS), olivetolic acid cyclase (OAC), prenyltransferase 4 (PT4; geranylpyrophosphate:olivetolate geranyltransferase), cannabidiolic acid synthase (CBDA), tetrahydrocannabinolic acid synthase (THCA), cannabichromenic acid synthase (CBCA)*.

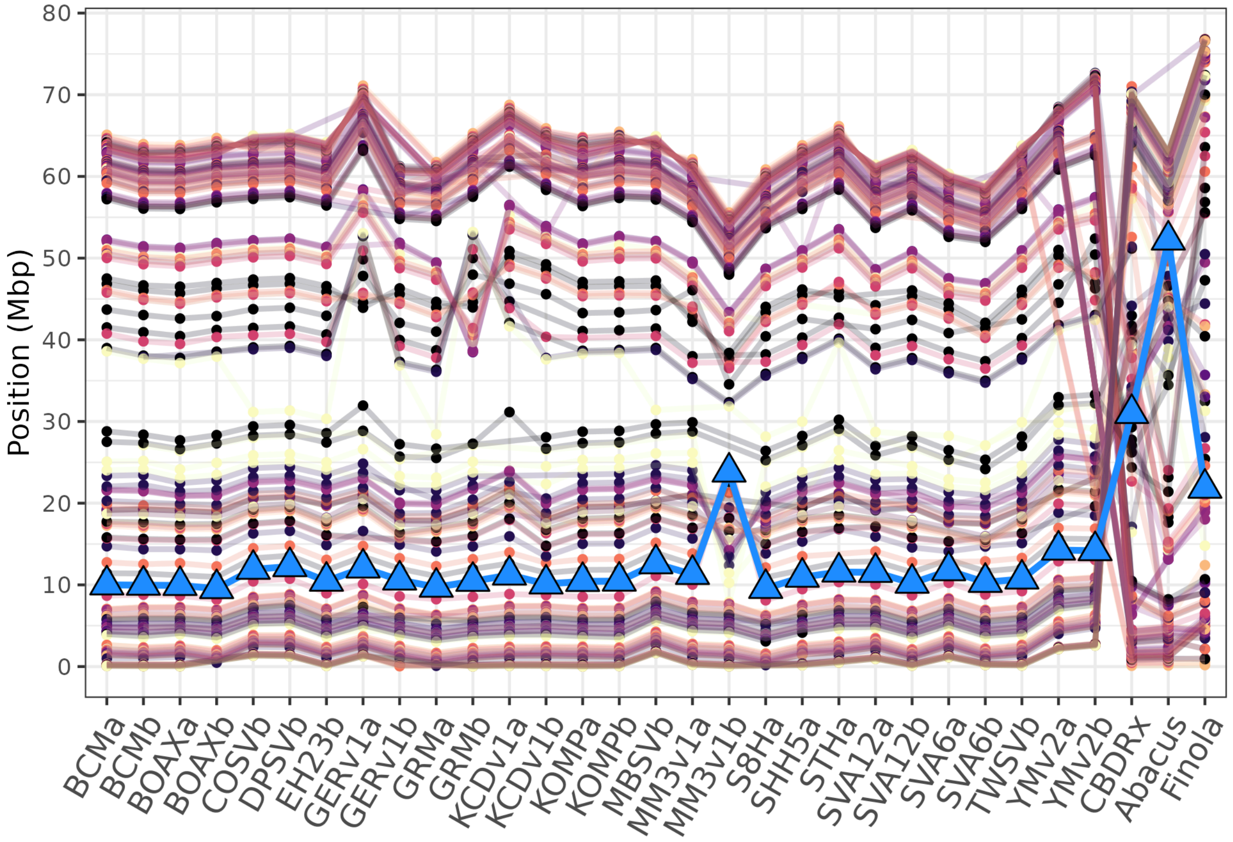

Supplementary Fig. 20. Comparison of BUSCO location and organization for a subset of scaffolded assemblies. Points and lines indicate the location of BUSCO genes (y-axis) on different chromosomes (x-axis). The location of *CBDAS* (>= 98% identity to AB292682.1) is denoted by the blue triangles and line. The position of *CBDAS* in CBDRx is approximately 30 Mb, in the pangenome assemblies this gene is consistently located at ~10 Mb, which suggests that *CBDAS* is misplaced in CBDRx.

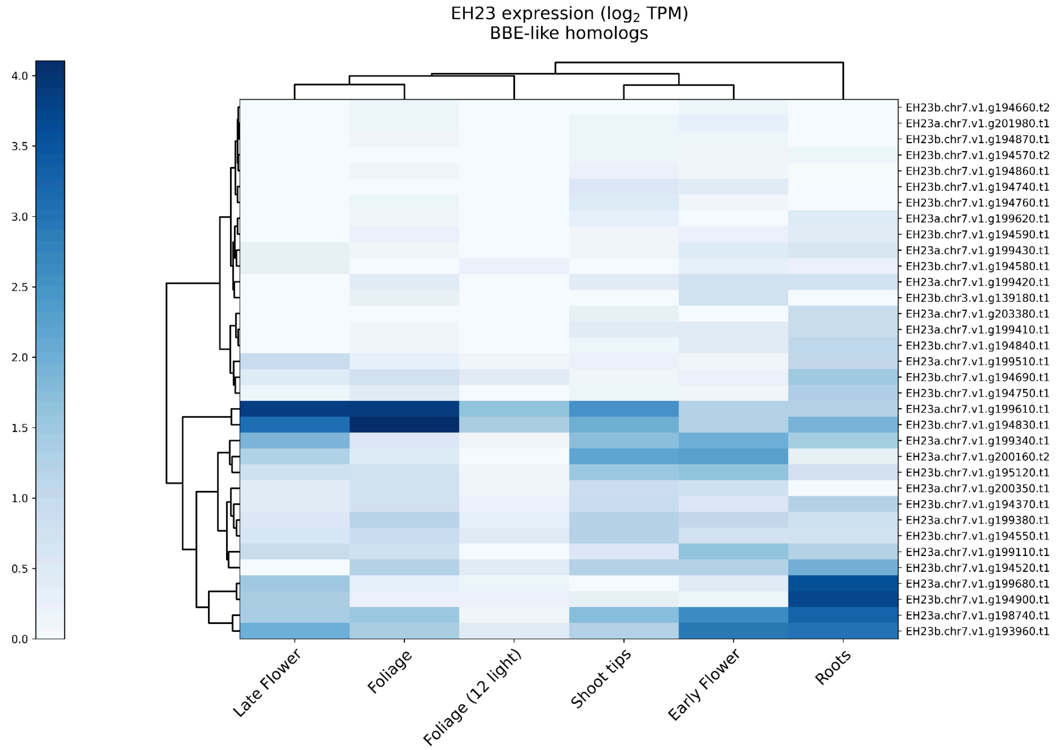

Supplementary Fig. 21. Expression of berberine bridge enzyme (BBE)-like genes in EH23. BBE-like genes are typically involved in alkaloid biosynthesis and are thought to be the ancestral gene of the cannabinoid synthases [9], both having an FAD-binding domain. BBE-like genes are almost exclusively expressed on chromosome 7, with foliage, late-stage flower, and roots showing highest expression.

Supplementary Fig. 22. Cannabinoid synthase cassettes in all assemblies grouped by shape and organization. Overall cassette shape is consistent for each of the full length synthases (*CBDAS, CBCAS, THCAS*), with moderate variation in the number of partial synthase copies. Full length *CBDAS* and *THCAS* occur as single copies in all haploid genomes. However, among the type II genomes, which contain CBDA and THCA synthases in separate haplotypes, the cassette shapes follow type III and type I cassette organization, respectively. In contrast, *CBCAS* has the greatest variation in the number of full length synthases. Full length *CBCAS* paralogs were typically located 15-20 Mb closer to the chr7 centromere, but due to inversion were sometimes found within ~1.2 Mb of *THCAS* (Fig. 4d). Across the pangenome *CBCAS* were present in 56% (110/193) of genomes, and were found in arrays of one to 15 copies. While it was shown that *CBCAS* genes can produce CBCA *in vivo* when transformed into yeast [10], an analysis of over 59,000 cannabis samples found effectively no evidence for CBCA content, although limited commercial testing for CBCA may have led to an underestimation [11]. Likewise, for the three *CBCAS* gene models in EH23, RNA expression was very low (at most ~1.7 TPM in shoot tissue), suggesting high levels of CBCA have not been selected for, perhaps due to human preference for THC and CBD. Examining the upstream regions of the synthases, Helitron DNA TEs and LTR-RTs were significantly enriched within 2 kb of *THCAS* genes (Supplementary Table 14), which may underlie gene duplication and evolution of promoters, similar to maize and other plants [12]. Since helitron and LTR-RT sequences upstream of the cannabinoid synthases clustered largely according to their corresponding synthase type [13], but show more diversity than synthase genes (Fig. 4e, Supplementary Fig. 31), improvements to the

CBCAS specific promoters could provide the key to unlocking a novel CBCA dominant chemotype. Link to high-resolution figure on figshare: **https://doi.org/10.6084/m9.figshare.25872250.v1**.

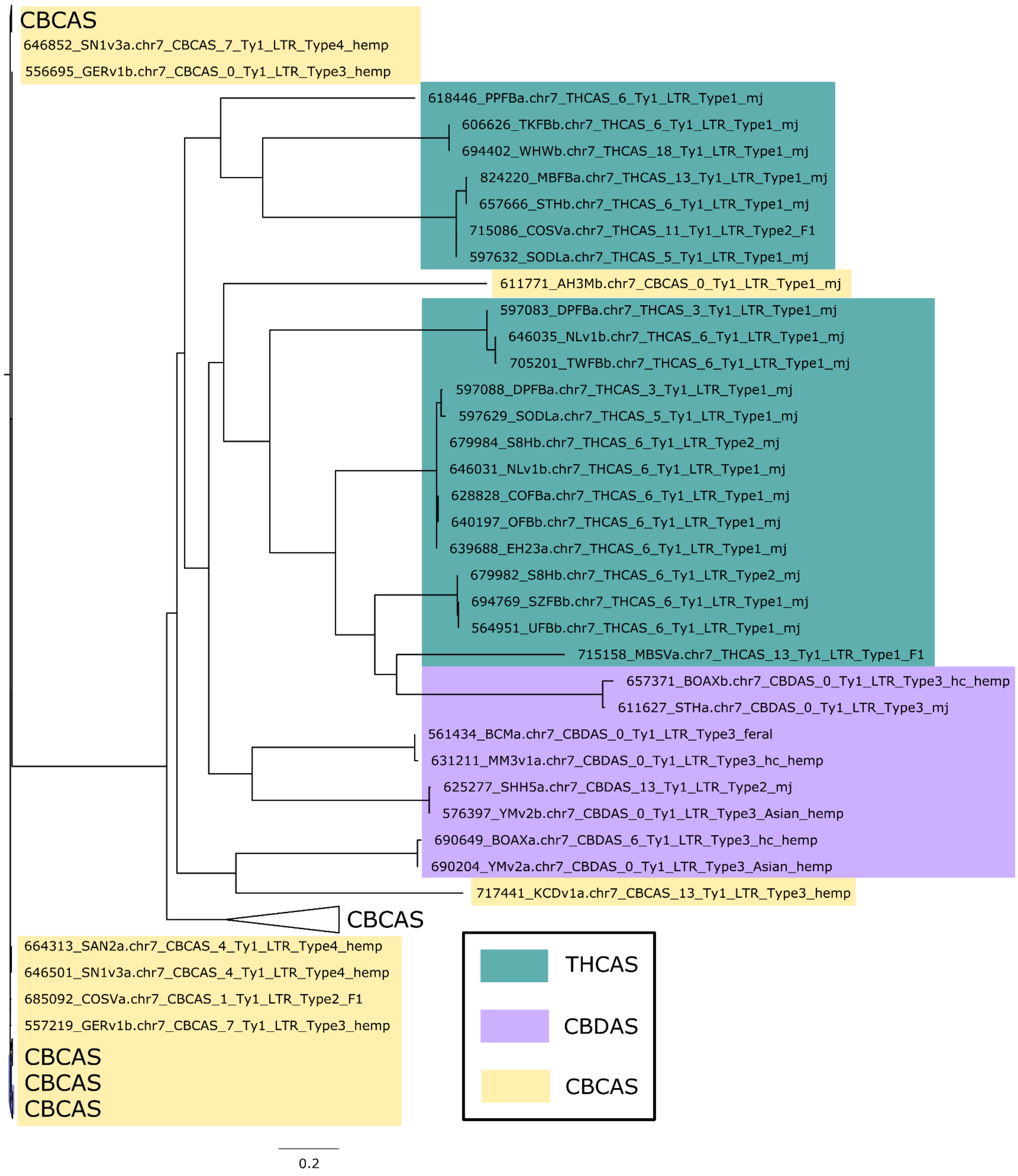

Supplementary Fig. 23. Maximum likelihood tree of Ty1 LTR-RT sequences flanking (2 kb upstream or downstream) cannabinoid synthases in the 78 scaffolded assemblies. Proximal LTR-RT sequences group largely according to full length cannabinoid synthase.

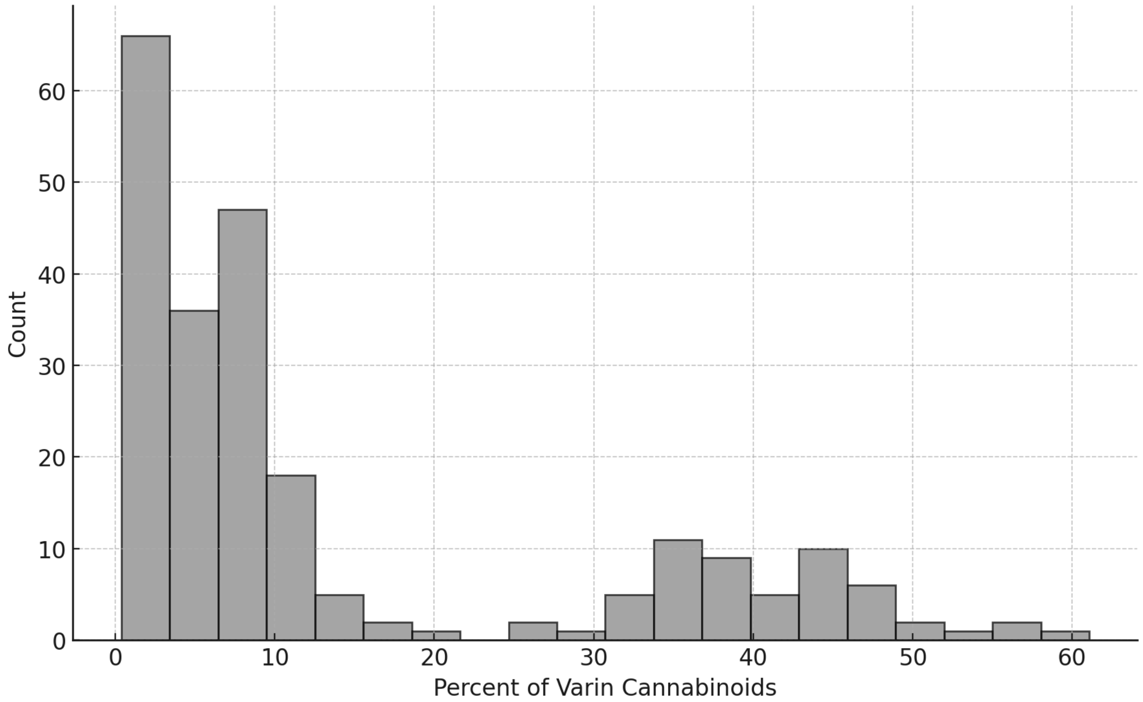

Supplementary Fig. 24. Percent of varin (propyl) cannabinoid (of total cannabinoids produced) histogram of F2 population (n = 318).

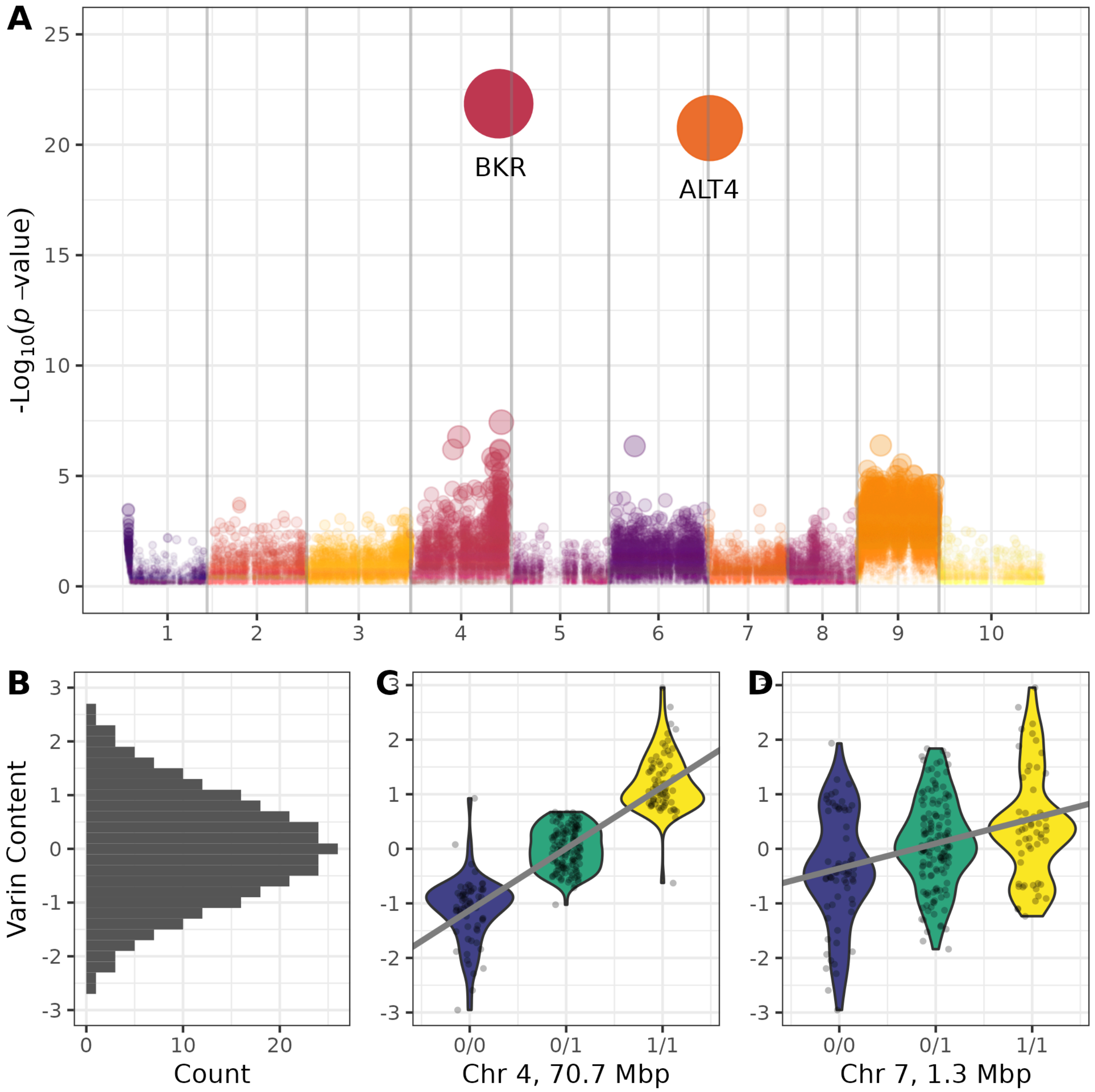

Supplementary Fig. 25. Varin (propyl) cannabinoid ratio GWAS. A) Significant GWA markers, using the BLINK model, 6 principal components, and order-normalized varin cannabinoid data. B) The distribution of propyl cannabinoid for the F2 population, where count is the number of plants measured. C) Phenotypic variance explained by a marker on chromosome 4, position 70,745,833 bp (r = 0.86, t = 27.668, df = 268, p-value < 2.2e-16). D) Phenotypic variance explained by a marker on chromosome 7, position 1,328,482 bp (r = 0.28, t = 4.8207, df = 268, p-value = 2.399e-06).

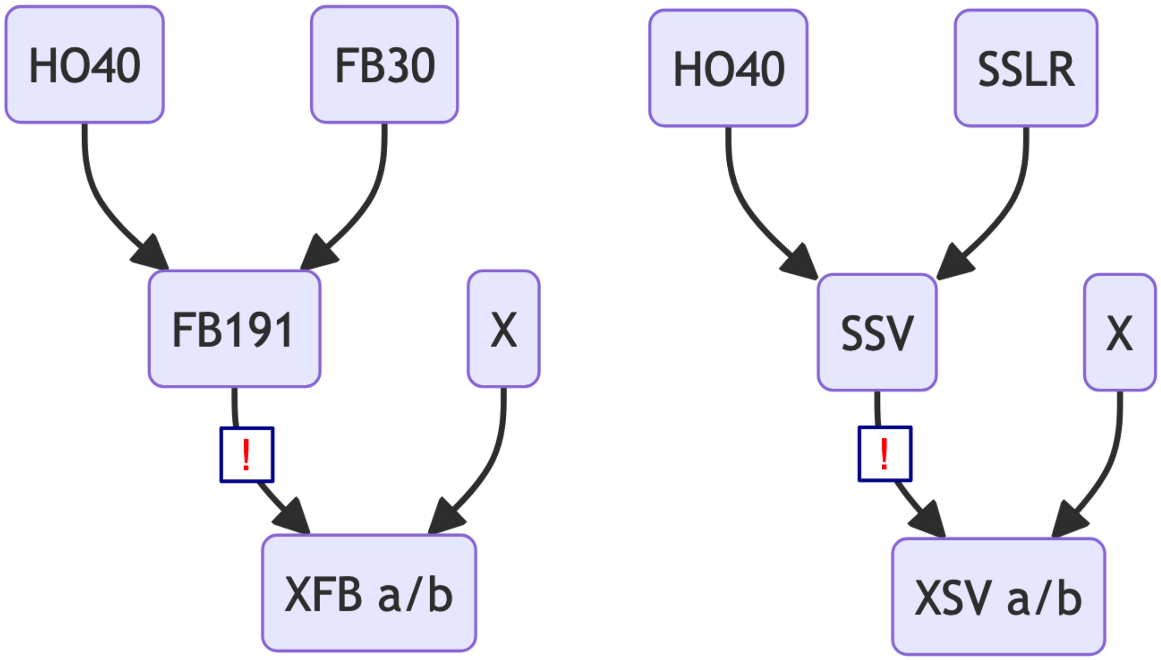

Supplementary Fig. 26. Cannabis trios. This figure shows the samples that were used in the PanKmer crossover analysis and how they were related to the original high varin plant, HO40. FB191 and SSV were crosses of HO40 with another parent (denoted as “X”). The red exclamation point corresponds to the samples carrying the varin locus. The subsequent generations (XFBa/b and XSVa/b) were included in the pangenome as haplotype-resolved assemblies. This figure accompanies Supplementary Table 15.

Supplementary Tables

Supplementary Table 1. Pangenome sample and assembly details: https://doi.org/10.6084/m9.figshare.25869319

Supplementary Table 2. Support for gene models, which were predicted based on homology and expression data from flowers, leaves, and roots using the TSEBRA pipeline (using Braker v2.1.6) [14] and eggnog-mapper (v2.0.1) [15].

Cultivar	Tissue	Sequencing method	NCBI BioProject
Sour Diesel, Canna Tsu, Black Lime, Valley Fire, White Cookies, Mama Thai, Terple, Cherry Chem, Blackberry Kush	Flower	Short-read Illumina	PRJNA498707
ERB x H040 (EH23)	Roots, foliage, foliage collected at 12 hours lights on/12 hours lights off, shoot tips, early-stage flower, late-stage flower	Short-read Illumina	Pangenome release
Fortune Cookie x Santhica 27 (FC6xSan27, FCS1)	Flower (collected at 5:00AM, 8:00AM, 3:00PM, 8:00PM),<br><br>Leaf (collected at 5:00AM, 8:00AM, 3:00PM, 8:00PM)<br><br>Stem (collected at 5:00AM, 8:00PM)<br><br>Small roots (collected at 8:00AM)<br><br>Tap root (collected at 8:00AM)	Full-length Oxford Nanopore cDNA	Pangenome release
Hercules x Santhica 27 (H35xSan27, H3S1)	Flower (collected at 5:00AM, 8:00AM, 8:00PM)<br><br>Leaf (collected at 5:00AM, 8:00AM, 3:00PM, 8:00PM)	Full-length Oxford Nanopore cDNA	Pangenome release

	Small roots (collected at 8:00AM)<br><br>Tap root (collected at 8:00AM)		
Santhica 27 (SAN2)	Flower (collected at 8:00AM [2 reps], 8:00PM)<br><br>Leaf (collected at 5:00AM, 8:00PM)<br><br>Stem (collected at 8:00PM)<br><br>Small roots (collected at 8:00AM)<br><br>Tap root (collected at 8:00AM)	Full-length Oxford Nanopore cDNA	Pangenome release

Supplementary Table 3. Balanced and biased gene expression in EH23.

	Early Flower	Foliage	Foliage 12 light	Late Flower	Roots	Shoot Tips
Total (1:1 Gene Pairs)	22426	22426	22426	22426	22426	22426
Balanced(#)	21302	21298	21725	20983	21527	21183
Balanced(%)	94.99	94.97	96.87	93.57	95.99	94.46
EH23a(#)	548	554	351	722	456	596
EH23a(%)	2.44	2.47	1.57	3.22	2.03	2.66
EH23b(#)	576	574	350	721	443	647
EH23b(%)	2.57	2.56	1.56	3.22	1.98	2.89

Supplementary Table 4. This table reports the most common GO terms that are significantly enriched among core genes with the 10% highest frequency among the 193 genomes. GO term enrichment was first evaluated for each genome separately, and then common GO terms were collected.

**GO ID**	**Number of genomes with significant GO term**	**GO Term**

GO:0051762	192	sesquiterpene biosynthetic process
GO:0045338	190	farnesyl diphosphate metabolic process
GO:0043693	186	monoterpene biosynthetic process
GO:2000023	184	regulation of lateral root development
GO:0006342	183	chromatin silencing
GO:0016099	182	monoterpenoid biosynthetic process
GO:0009684	182	indoleacetic acid biosynthetic process
GO:0034220	179	ion transmembrane transport
GO:0016106	177	sesquiterpenoid biosynthetic process
GO:0009626	172	plant-type hypersensitive response
GO:0080027	170	response to herbivore
GO:0019745	167	pentacyclic triterpenoid biosynthetic process
GO:0048864	166	stem cell development
GO:0010042	164	response to manganese ion
GO:0009753	162	response to jasmonic acid
GO:0046244	158	salicylic acid catabolic process
GO:0009739	154	response to gibberellin
GO:0010233	145	phloem transport
GO:0010043	134	response to zinc ion
GO:0007165	133	signal transduction
GO:0070476	132	rRNA (guanine-N7)-methylation
GO:0048527	131	lateral root development
GO:0009555	130	pollen development
GO:0006949	127	syncytium formation
GO:0055085	126	transmembrane transport
GO:0019756	125	cyanogenic glycoside biosynthetic process
GO:0043473	125	pigmentation
GO:0016131	119	brassinosteroid metabolic process
GO:0046323	114	glucose import

GO:0006686	113	sphingomyelin biosynthetic process
GO:1904504	113	positive regulation of lipophagy
GO:0046511	113	sphinganine biosynthetic process
GO:0009625	111	response to insect
GO:0032197	109	transposition RNA-mediated
GO:0002229	107	defense response to oomycetes
GO:0006278	107	RNA-dependent DNA biosynthetic process
GO:0015692	102	lead ion transport
GO:0046686	99	response to cadmium ion
GO:0010224	93	response to UV-B
GO:0016598	92	protein arginylation
GO:0030639	87	polyketide biosynthetic process
GO:0016572	85	histone phosphorylation

Supplementary Table 5. Pairwise *Fst* values (Weir and Cockerham weighted) for the populations based on SNPs.

**Comparison**	**weighted F_{st}**
mj vs feral	0.21
asian hemp vs mj	0.21
EU_hemp vs mj	0.20
asian hemp vs F1s	0.15
EU_hemp vs asian hemp	0.14
F1s vs feral	0.13
EU_hemp vs hc hemp	0.10
EU_hemp vs F1s	0.10
mj vs hc hemp	0.07
asian hemp vs hc hemp	0.05
EU_hemp vs feral	0.04
hc hemp vs F1s	0.04
mj vs F1s	0.04

hc hemp vs feral	0.03

Supplementary Table 6. High F_{st} genes that are linked to environmental response, with circadian, light signaling, and flowering time genes exhibiting an above-average *Fst* (0.42). The full F_{st} and XP-CLR results are available from Figshare**.**

Fst	**gene ID**	**name**	**type**
0.854823	EH23a.chr9.v1.g267660.t1	BHLH92	clock
0.848677	EH23a.chr2.v1.g111360.t1	PIF3	light
0.848403	EH23a.chr2.v1.g111160.t1	PHYA	light
0.821622	EH23a.chr2.v1.g083400.t1	RVE8	clock
0.784696	EH23a.chr3.v1.g139280.t1	RVE4	clock
0.781328	EH23a.chr2.v1.g090180.t1	MED14 (next to FLK)	development/thermomorphogenesis
0.779475	EH23a.chrX.v1.g343490.t1	GI	clock
0.718032	EH23a.chr7.v1.g196830.t1	DDB1A	light
0.711334	EH23a.chr4.v1.g171380.t1	UVR8	light
0.711334	EH23a.chr7.v1.g196420.t1	PTAC12/HMR	light
0.703466	EH23a.chrX.v1.g336130.t1	FIO1	clock
0.700321	EH23a.chr2.v1.g113520.t1	PHOT1	light
0.700321	EH23a.chr6.v1.g290020.t1	SRT1	development
0.700321	EH23a.chr8.v1.g228120.t1	SPY	flowering
0.627913	EH23a.chr6.v1.g291390.t1	ZTL	clock
0.627913	EH23a.chr7.v1.g194090.t1	PHYE	light
0.627913	EH23a.chr9.v1.g256130.t1	CRY2	light
0.625187	EH23a.chr2.v1.g087970.t1	HY2	light
0.61286	EH23a.chr1.v1.g022720.t1	SPA1	light
0.61286	EH23a.chr2.v1.g112090.t1	CRB	clock
0.612158	EH23a.chr1.v1.g037900.t1	PRR3	clock
0.612158	EH23a.chr3.v1.g131990.t1	PIF7	light
0.612158	EH23a.chr4.v1.g155590.t1	MED23a	development
0.612158	EH23a.chr8.v1.g231480.t1	PIE1	flowering

0.612158	EH23a.chrX.v1.g325410.t1	PHOT2	light
0.596154	EH23a.chr4.v1.g190350.t1	PIL5	light
0.596154	EH23a.chr8.v1.g238790.t1	BBX8	clock
0.589987	EH23a.chr8.v1.g246800.t1	TIR3/BIG	phytohormone
0.569224	EH23a.chr9.v1.g267720.t1	TZP	light
0.562834	EH23a.chr7.v1.g196430.t1	FTa	flowering
0.560587	EH23a.chr3.v1.g141480.t1	PRMT5	clock
0.526007	EH23a.chr9.v1.g264180.t1	MED33A	development
0.513716	EH23a.chr4.v1.g155960.t1	FKF1	clock
0.512419	EH23a.chrX.v1.g348580.t1	CBK4	clock
0.487618	EH23a.chr1.v1.g040370.t1	ELF4	clock
0.487618	EH23a.chr2.v1.g085990.t1	TEJ	clock
0.487618	EH23a.chr5.v1.g079470.t1	PRR5	clock
0.487618	EH23a.chr8.v1.g242990.t1	COP1	light
0.487618	EH23a.chr8.v1.g245460.t1	LIP1	clock
0.487618	EH23a.chr6.v1.g321750.t1	FCA	flowering
0.45048	EH23a.chr7.v1.g196700.t1	PID	flower morph
0.40208	EH23a.chr9.v1.g256870.t1	BRK1	phytohormone
0.398717	EH23a.chr8.v1.g234170.t1	MED25/PFT1	development/flowering
0.375215	EH23a.chr1.v1.g037200.t1	MED4	development
0.367701	EH23a.chr1.v1.g003070.t1	UVR3	light
0.367701	EH23a.chr3.v1.g130580.t1	CDF3	clock
0.367701	EH23a.chr5.v1.g073540.t1	RVE5	clock
0.367701	EH23a.chr6.v1.g292420.t1	BBX6/COL5	flowering
0.367701	EH23a.chrX.v1.g337260.t1	RTV1	vernalization
0.336321	EH23a.chrX.v1.g328670.t1	EID1	light
0.322581	EH23a.chr3.v1.g132760.t1	TOC1	clock
0.322581	EH23a.chr5.v1.g070330.t1	TCP	clock
0.322581	EH23a.chr9.v1.g257470.t1	BRK1	phytohormone

0.322581	EH23a.chr1.v1.g036320.t1	HUA1	development
0.30363	EH23a.chr7.v1.g196580.t1	SRR1	light
0.293593	EH23a.chr8.v1.g244330.t1	PIL6	light
0.282997	EH23a.chr1.v1.g024760.t1	LHY	clock
0.282997	EH23a.chr8.v1.g234410.t1	STN7	light
0.274902	EH23a.chr8.v1.g239870.t1	FHY3	light
0.261557	EH23a.chr8.v1.g246500.t1	CO	flowering
0.261557	EH23a.chr9.v1.g261160.t1	PHYB	light
0.258543	EH23a.chr7.v1.g219420.t1	MED16	development
0.199112	EH23a.chr4.v1.g157580.t1	MED23b	development
0.181058	EH23a.chr3.v1.g144180.t1	CRY3	light
0.181058	EH23a.chr4.v1.g172720.t1	DET1	light
0.181058	EH23a.chr4.v1.g175090.t1	MED23c	development
0.181058	EH23a.chr4.v1.g183760.t1	SPT	development
0.181058	EH23a.chr9.v1.g265260.t1	TIC	clock
0.168277	EH23a.chr2.v1.g090690.t1	FLK	flowering
0.161278	EH23a.chr1.v1.g024530.t1	PRR9	clock
0.144269	EH23a.chrX.v1.g335960.t1	XCT	clock
0.125295	EH23a.chrX.v1.g365010.t1	CRY1	light
0.122451	EH23a.chr4.v1.g164880.t1	EPR1/RVE7a	clock
0.104365	EH23a.chr2.v1.g105740.t1	PCL1	clock
0.0814438	EH23a.chr6.v1.g315450.t1	LNK1	clock
0.0635788	EH23a.chr1.v1.g037760.t1	TOE	flowering
0.0437042	EH23a.chr8.v1.g255440.t1	FTb	flowering
0.0241636	EH23a.chrX.v1.g338050.t1	ELF3	clock
0.0168509	EH23a.chr4.v1.g164900.t1	EPR1/RVE7b	clock
0.0168509	EH23a.chr8.v1.g247990.t1	LWD1	clock
0.0168509	EH23a.chr7.v1.g202320.t1	AGO3	epigenetics
0.0150034	EH23a.chr6.v1.g291700.t1	VIN3	vernalization

-0.050821	EH23a.chrX.v1.g354800.t1	HY5	light

Supplementary Table 7. Syntenic orthogroup (OG) counts in genomes with X and Y sex chromosomes (including AH3M, BCM, GRM, KOMP) based on genespace analysis (see also Supplementary Figure 15).

Genome set	Syntenic OGs found in all eight genomes	Syntenic OGs in male autosomes	Syntenic OGs in female autosomes	Syntenic OGs present in both X and Y chromosomes	Syntenic OGs present only in X chromosomes	Syntenic OGs present only in Y chromosomes
Eight genomes with X and Y chromosomes	15,127	7	18	1,371	1,191	428

Supplementary Table 8. Genomic coordinates for SDR (sex-determining region)-PAR (pseudoautosomal region) boundary.

Chromosome	Start	Stop	Region	Strand
AH3Ma.chrX	54629256	84231629	PAR	-
AH3Ma.chrX	1	54629255	X_specific_region	-
AH3Mb.chrY	1	29081709	PAR	+
AH3Mb.chrY	29081710	110682302	SDR	+
BCMa.chrX	1	28914799	PAR	+
BCMa.chrX	28914800	83331736	X_specific_region	+
BCMb.chrY	1	28433130	PAR	+
BCMb.chrY	28433131	107756508	SDR	+
GRMa.chrY	83638349	113162000	PAR	-
GRMa.chrY	1	83638348	SDR	-
GRMb.chrX	1	29140387	PAR	+
GRMb.chrX	29140388	83804036	X_specific_region	+
KOMPa.chrX	52909065	84504832	PAR	-
KOMPa.chrX	1	52909064	X_specific_region	-
KOMPb.chrY	1	30469916	PAR	+

KOMPb.chrY	30469917	110614000	SDR	+

Supplementary Table 9. Average percent of scaffolded assemblies covered by transposable elements identified with EDTA. See also Supplementary Table 1 under the tab "ASSEMBLY_FINAL" for percentages from RepeatMasker.

Transposable element category	Average repeat content (%)	Standard deviation (%)
Total repeat content	67.89	1.19
Ty1 LTR-RT	16.27	1.68
Ty3 LTR-RT	19.7	1.32
Unknown LTR-RT	16.51	2.46
CACTA	3.12	1.59
Harbinger	1.09	0.32
hAT	1.95	1.21
helitron	2.84	1.68
Mariner	0.37	0.55
Mutator	6.03	1.45

Supplementary Table 10. Average distance between genes and transposable elements in scaffolded assemblies.

Transposable element category	Median distance (bp)	Average distance (bp)	Standard deviation (bp)
Ty1 LTR-RT	238	491.91	840.17
Ty3 LTR-RT	138	476.49	968.93
Unknown LTR-RT	279	538.85	1251.94
CACTA	103	443.83	940.63
Harbinger	335	551.38	916.6
hAT	359	613.82	1025.01
Helitron	312	549.16	890.42
Mariner	142	506.79	1270.51
Mutator	274	517.64	897.37

Supplementary Table 11. Functional enrichment of genes located near TEs across the full pangenome.

GO ID	GO Term	Number of genomes with significant GO term
	**CACTA**	
GO:0006355	regulation of transcription, DNA-templated	17
GO:0048864	stem cell development	8
GO:0009269	response to desiccation	6
GO:0030154	cell differentiation	5
	**Ty1-LTR retrotransposon**	
GO:1900706	positive regulation of siderophore biosynthetic process	16
GO:0071731	response to nitric oxide	15
GO:0010104	regulation of ethylene-activated signaling pathway	15
GO:0046685	response to arsenic-containing substance	13
GO:1990641	response to iron ion starvation	11
GO:0006468	protein phosphorylation	11
GO:0010106	cellular response to iron ion starvation	8
GO:0007165	signal transduction	8
GO:0051762	sesquiterpene biosynthetic process	7
GO:0006357	regulation of transcription by RNA polymerase II	7
GO:0010073	meristem maintenance	7
GO:0009414	response to water deprivation	7

GO:0055114	oxidation-reduction process	6
GO:0010501	RNA secondary structure unwinding	6
GO:0006355	regulation of transcription, DNA-templated	6
GO:0006177	GMP biosynthetic process	6
GO:0009992	cellular water homeostasis	6
GO:0006855	drug transmembrane transport	5
GO:0009311	oligosaccharide metabolic process	5
**Ty3-LTR retrotransposon**		
GO:0032197	transposition, RNA-mediated	156
GO:0006278	RNA-dependent DNA biosynthetic process	150
GO:0090501	RNA phosphodiester bond hydrolysis	110
GO:0006508	proteolysis	94
GO:0090378	seed trichome elongation	20
GO:0006355	regulation of transcription, DNA-templated	20
GO:0006338	chromatin remodeling	10
GO:0000209	protein polyubiquitination	9
GO:0006177	GMP biosynthetic process	8
**hAT**		
GO:0009812	flavonoid metabolic process	6
GO:0006890	retrograde vesicle-mediated transport, Golgi to ER	6
**Helitron**		

GO:0048658	anther wall tapetum development	8
GO:0010623	programmed cell death involved in cell development	7
**Mutator**		
GO:0009651	response to salt stress	15
GO:0006355	regulation of transcription, DNA-templated	13
GO:0055114	oxidation-reduction process	9
GO:0080167	response to karrikin	7
GO:0009414	response to water deprivation	7
GO:0006096	glycolytic process	5
GO:0009789	positive regulation of abscisic acid-activated signaling pathway	5
GO:0043161	proteasome-mediated ubiquitin-dependent protein catabolic process	5
GO:0010431	seed maturation	5
GO:0009793	embryo development ending in seed dormancy	5
**Harbinger**		
GO:0019756	cyanogenic glycoside biosynthetic process	6
GO:0009684	indoleacetic acid biosynthetic process	6
GO:0098542	defense response to other organism	5
GO:0009821	alkaloid biosynthetic process	5

Supplementary Table 12. Average methylation (%) across the genome.

Genome	Population information	Mean methylation level (%) across the genome in haplotype A	Mean methylation level (%) across the genome in haplotype B
AH3M	MJ, type 1	84.37	87.47
KCDv1	Hemp, type 3	83.35	84.37
NLv1	MJ, type 1	84.36	87.10
WHW	MJ, type 1	79.64	81.65
SODL	MJ, type 1	84.45	85.73
SN1v3	Hemp, type 4	73.82	80.36
SAN2	Hemp, type 4	73.10	80.04
FCS1	F1, type 1 (FCS1a), type 4 (FCS1b)	78.0	82.10
H3S1	F1, type 1 (H3S1a), type 4 (H3S1b)	80.33	82.99

Supplementary Table 13. Number of TEs associated with breakpoints of SVs (duplications, inversions, inverted translocations, and translocations). The observed TE count is the total number of TEs associated with a given SV among the genomes in each population. The average bootstrapped TE count (1,000 bootstrap replicates) is the total number of TEs associated with a random region of the genome that has the same length and is from the same chromosome as an observed SV. A positive test statistic indicates TE enrichment and negative test statistic indicates TE depletion. This analysis includes the 78 chromosome-level, haplotype-resolved genomes.

Duplications						
**popID**	**featureID**	**Observed TE counts**	**Average bootstrapped TE counts**	**Average bootstrapped test statistic**	**Average bootstrapped P value**	**Average bootstrapped, corrected P value**
mj	Ty3_LTR-RT	6335	6073.95	3.71	2.29E-03	2.52E-03
mj	Mutator_TIR_transposon	11788	7632.38	19.75	7.84E-66	7.84E-66
mj	PIF_Harbinger_TIR_transposon	2000	1499.82	4.84	1.22E-05	1.37E-05
mj	hAT_TIR_transposon	4574	2389.23	20.45	1.05E-70	1.06E-70
mj	helitron	767	909.93	-4.52	1.18E-04	1.28E-04
hc_hemp	Mutator_TIR_transposon	752	627.62	3.45	6.87E-03	7.59E-03

F1	Mutator_TIR_transposon	8667	7639.45	6.06	8.29E-07	8.41E-07
F1	helitron	171	230.15	-4.01	1.06E-03	1.16E-03
F1	PIF_Harbinger_TIR_transposon	683	517.23	5.17	3.39E-05	3.51E-05
hemp	Mutator_TIR_transposon	2833	1789.29	9.66	9.95E-16	9.96E-16
hemp	PIF_Harbinger_TIR_transposon	966	680.07	9.46	4.45E-14	4.46E-14
Asian_hemp	Mutator_TIR_transposon	2503	1563.51	8.66	1.46E-11	1.46E-11
**Inversions**						
**popID**	**featureID**	**Observed TE counts**	**Average bootstrapped TE counts**	**Average bootstrapped test statistic**	**Average bootstrapped P values**	**Average bootstrapped, corrected P values**
mj	Ty1_LTR-RT	2378	2167.67	3.77	3.07E-03	3.30E-03
mj	Mutator_TIR_transposon	803	614.02	3.83	2.37E-03	2.57E-03
mj	helitron	590	444.6	4.48	2.55E-04	2.69E-04
F1	hAT_TIR_transposon	170	106.43	3.73	5.84E-03	6.33E-03
F1	helitron	192	124.82	3.34	6.05E-03	7.02E-03
**Inverted translocations**						
**popID**	**featureID**	**Observed TE counts**	**Average bootstrapped TE counts**	**Average bootstrapped test statistic**	**Average bootstrapped P values**	**Average bootstrapped, corrected P values**
mj	Mutator_TIR_transposon	3417	2624.54	7.02	6.77E-08	6.78E-08
mj	Ty1_LTR-RT	6917	6626.22	3.60	3.76E-03	4.14E-03
mj	hAT_TIR_transposon	1131	816.52	5.41	6.72E-06	6.93E-06
mj	PIF_Harbinger_TIR_transposon	680	477.89	6.00	9.55E-07	9.79E-07
feral	Mutator_TIR_transposon	369	255.19	3.28	1.85E-03	3.17E-03
hc_hemp	Ty3_LTR-RT	1815	1689.54	3.66	2.17E-03	2.45E-03
hc_hemp	Mutator_TIR_transposon	517	357.22	3.44	5.61E-03	6.35E-03
F1	PIF_Harbinger_TIR_transposon	228	136.36	4.47	7.42E-04	7.76E-04
F1	Mutator_TIR_transposon	1916	1362.6	6.42	7.67E-07	7.68E-07

Asian_hemp	Mutator_TIR_transposon	553	289.07	5.43	4.31E-05	4.44E-05
**Translocations**						
**popID**	**featureID**	**Observed TE counts**	**Average bootstrapped TE counts**	**Average bootstrapped test statistic**	**Average bootstrapped P values**	**Average bootstrapped, corrected P values**
mj	hAT_TIR_transposon	1314	832.38	7.97	2.80E-11	2.81E-11
mj	Mutator_TIR_transposon	3936	2691.91	10.57	3.49E-18	3.49E-18
mj	unknown_LTR	7888	7489.27	3.57	4.10E-03	4.54E-03
mj	Ty3_LTR	6729	6386.75	4.82	9.33E-05	9.63E-05
mj	PIF_Harbinger_TIR_transposon	680	441.16	5.86	2.39E-05	2.41E-05
hc_hemp	Mutator_TIR_transposon	644	421.97	3.89	2.26E-03	2.44E-03
F1	Mutator_TIR_transposon	1584	1299.08	3.44	4.07E-03	4.69E-03
F1	PIF_Harbinger_TIR_transposon	313	159.77	6.44	1.76E-07	1.79E-07
hemp	Mutator_TIR_transposon	970	759.26	3.40	4.85E-03	5.55E-03
Asian_hemp	Mutator_TIR_transposon	620	398.07	3.64	3.70E-03	4.13E-03

Supplementary Table 14. Statistically significant TEs upstream (2 kb) and downstream (2 kb) of cannabinoid synthases. A specific category of TE is denoted as "X," and "Y" includes all types of TEs. The total number of X TEs located upstream or downstream of a cannabinoid synthase is denoted as "a"; the total number of "X" TEs located upstream or downstream of any gene is "b"; the total number of any TE located upstream or downstream of a cannabinoid synthase is "c"; and the total number of "Y" TEs located upstream or downstream of any gene is "d." An enrichment score (ES) is defined as $ES = (a/b)/(c/d)$, and the p-value is defined as $p = (a + b)!\,(c + d)!\,(a + c)!\,(b + d)!\,/(a!\,b!\,c!\,d!\,N!)$, where N is the sum of a, b, c, and d. See section "**Enrichment of transposable elements flanking genomic features**" in the Supplementary Material for additional detail.

**Flanking (2 kb)**	**Molecule ID**	**TE Feature**	**a**	**b**	**c**	**d**	**Enrichment score**	**P-value**	**FDR**
upstream	CBCAS	Unknown LTR-RT fragment	326	450447	645	6232481	6.99	1.53E-134	2.91E-133
upstream	CBCAS	Ty3 LTR-RT fragment	222	285594	645	6232481	7.51	4.13E-102	3.93E-101
upstream	CBDAS	Unknown LTR-RT internal	40	786199	76	6232481	4.17	2.48E-11	7.84E-11
upstream	THCAS	Helitron fragment	45	717582	126	6232481	3.1	2.01E-09	5.45E-09
upstream	THCAS	Ty1 LTR-RT internal	33	710906	126	6232481	2.3	3.83E-05	7.27E-05

upstream	THCAS	Unknown LTR-RT internal	35	786199	126	6232481	2.2	5.19E-05	8.96E-05
downstream	CBCAS	Ty1 LTR-RT fragment	208	277130	407	3976423	7.33	6.93E-90	1.52E-88
downstream	THCAS	Unknown LTR-RT internal	53	454021	109	3976423	4.26	5.56E-15	4.08E-14
downstream	CBCAS	Ty1 LTR-RT internal	102	469415	407	3976423	2.12	1.17E-10	5.14E-10
downstream	THCAS	Ty3 LTR-RT internal	15	152242	109	3976423	3.59	4.21E-05	0.0001157 9
downstream	THCAS	Unknown LTR-RT fragment	23	341743	109	3976423	2.46	0.000172 55	0.0004217 9

Supplementary Table 15. Trios used in crossover analysis of varin. This table accompanies **Supplementary Figure 32**.

**Parent 1**	**Parent 2**	**Cross**	**Varin haplotype**
FB191	CaliO	COFB	a
.	Durban Poison	DPFB	b
.	Mendo Breath	MBFB	b
.	OFP	OFB	b
.	Purple Punch	PPFB	b
.	Sundae Driver	SDFB	b
.	Skunk 88	SKFB	b
.	Triangle Kush	TKFB	b
	Trainwreck	TWFB	b
.	Urkle	UFB	b
.	Wedding Cake	WCFB	b
SSV	CaliO	COSV	b
.	Durban Poison	DPSV	b
.	Mendo Breath	MBSV	b
.	SSA	SVA6	a
.	SSA	SVA12	a
	Trainwreck	TWSVb	b

Supplementary Table 16. Genetic variants significantly associated with varin cannabinoid ratio data (ordered quantile (ORQ) normalization transformed), using the BLINK model from GAPIT.

SNP	P-value	-Log10(p-value)	MAF	nobs	H.B.P.Value	Effect	Pos
EH23b.chr4_70745833	1.38E-22	21.86	0.491	270	1.29E-17	-1.056	70745833
EH23b.chr7_1328482	1.77E-21	20.75	0.478	270	8.23E-17	-0.358	1328482
EH23b.chr4_72901377	0.0000000367	7.44	0.489	270	0.00114	-0.596	72901377
EH23b.chr4_38647277	0.000000173	6.76	0.47	270	0.00403	0.432	38647277

Supplementary Note 1: EH23a and F2 mapping population

The EH23 high quality haplotype-resolved, chromosome-scale genome revealed differences in chromosome lengths, putative centromere regions, structural variation (SV), distinct locations of THCA and CBDA synthases, and gene densities (Fig.1a; Extended Data Figs. 3,5), in comparison with our other haplotype-resolved, chromosome-scale cannabis genomes (https://doi.org/10.6084/m9.figshare.25865188.v1, https://doi.org/10.25452/figshare.plus.28405079.v1). The abundance of the motif 'CpG' (cytosine, phosphate, guanine), which was correlated with methylation and centromere formation, varied throughout the chromosomes yet showed clear concentrations in gene-poor regions, consistent with a centromere signature. Additionally, we identified high copy number repeats consistent with centromere arrays: one was not found consistently across the chromosomes and the other was the sub-telomeric repeat [2] (Extended Data Figs. 5). Long telomere sequences (~50 Kb; AAACCCT) were found on all chromosome ends. The sub-telomeric repeat [2] was found on all but four chromosome ends (4/20) and was also found in the putative centromere (high CpG) regions in half (10/20) of the chromosomes (Fig. 1a; Extended Data Figs. 5).

An F2 population derived from selfing EH23 (ERBxHO40_23) exhibited mixed patterns of allele frequencies and F_{is} (deviation of heterozygosity from Hardy-Weinberg expectation) across the genome, likely the consequence of segregation distortion as seen in other cannabis crosses [10] (Extended Data Fig. 3). Segregation distortion patterns observed across many regions of the genome were noted in a prior study of this population [27], and may be caused by differences in seed germination rates. A study done by Beutler and Der Marderosian reported a delay in seed germination in crosses between day-neutral and short-day plants; the authors speculate this trait is a carryover from the hypothesized species "*Cannabis ruderalis*," or day-neutral, parent [28]. Alternatively, prezygotic selection such as meiotic drive in the female germline or pollen competition could drive deviations from Hardy-Weinberg equilibrium [29]. In *Arabidopsis* and rice many tested populations show segregation distortion, which may be due to seed dormancy and lethal epistatic interactions [30,31]. However, segregation distortion could be the result of SV noted across the pangenome. Consistent with this observation, day-neutral alleles have been mapped to chr1, which showed the largest size variation [27,32] (Extended Data Fig. 4).

The EH23 trio phased diploid F1 assembly provided the opportunity to evaluate haplotype specific expression patterns in cannabis. We sampled six tissue types from EH23 (shoot tips, roots, late flower, leaf under short-day photoperiodicity, leaf under long-day photoperiodicity, and early flower) to evaluate haplotype specific expression (Extended Data Fig. 3). Comparisons between tissue types showed that approximately equal percentages of transcripts were biased in either EH23a or EH23b (Extended Data Fig. 3), while most (~95% on average across tissues)

of the genes had balanced allele expression based on our criteria. Genes expressed specifically on the EH23a haplotype were found mostly on chr1 and X, while genes with biased expression on EH23b were mostly on chr1 and 4 (Extended Data Fig. 3).

Supplementary Note 2: Terpene and disease resistance genes

Terpenes are involved in a complex interplay of communication and defense against herbivores [16], as well as potentially modulating cannabinoid activity [17]. Terpenes in cannabis have been investigated previously [18–23], although the genomic organization and extent of variation of terpene synthases was not known. We found terpene synthase “hotspots” on chr5 and 6, where the majority of copies were located in a multi-megabase region on one end of the chromosome (Extended Data fig. 7, https://doi.org/10.6084/m9.figshare.25872136.v1), consistent with previous findings that chr5 and 6 have QTLs associated with consumer preference for “sweet” or “earthy” aroma profiles [19]. The majority of terpene synthase genes had seven exons, consistent with monoterpene or sesquiterpene biosynthesis [20].

In addition to primary gene annotations, we developed plant disease resistance gene analog (“R gene”) maps for the 78 scaffolded, haplotype-phased genomes using Drago2 (Extended Data fig. 8, https://doi.org/10.6084/m9.figshare.25872292.v1) [24]. Specific R genes encode protein functional domains that determine pathogen specificity and subcellular localization. In cannabis, the *MILDEW LOCUS O* (*MLO*) disease resistance gene family has received the most attention because of the widespread presence of powdery mildew disease caused by *Golvinomyces* spp. in commercial operations [25]. However, as the legal cannabis industry has expanded, new disease and pest challenges have emerged, including fungal pathogens such as *Fusarium spp.*, *Botrytis cinerea* and *Pythium* spp. [26]. Therefore, knowledge of the diversity of disease resistance genes and alleles has relevance to cannabis breeding. In the pangenome, receptor-like kinases (RLK) were the most abundant R-gene type and overall, R-genes were distributed across all chromosomes. The highest concentration and variation of R-genes was found in hotspots at the top of chr1 (Extended Data fig. 8). Most coiled-coil NBS-LRR genes (CNLs) were found on chromosomes 3 and 6. Together, these results uncovered genomic regions where specific disease resistance breeding targets could be developed (Extended Data fig. 8).

Supplementary Note 3: WGD analysis reveals cannabis has the eudicot hexaploidization

The high quality wild cannabis genome analysis provided evidence for a large whole genome duplication (WGD) or whole genome triplication (WGT) in the past, in addition to more recent WGD events [13]. We leveraged several chromosome-resolved haplotype genomes representing the different classes of cannabis and sexes to estimate the WGD history as well as whether the sex chromosomes were under different selection across the populations. First, we performed all-all protein alignments within and between cannabis genomes representing type I, type III, feral and fiber. All of the alignments resulted in a syntenic depth of 1:1 with off-diagonal patterns more consistent with unmasked transposable elements (TEs) remaining in the genomes (Supplementary Fig. 6). We estimated the synonymous substitution (ks) rate between syntenic proteins to determine when genomes diverged, and found that self-self and haplotype-haplotype cannabis alignments revealed early peaks around 1 and 10 million years ago (MYA) respectively (Supplementary Fig. 6). However, consistent with the syntenic depth analysis, the gene pairs driving the early peak were unmasked TEs. When the unmasked TEs were removed, the Ks distribution suggested genes off the diagonal were duplicated around 100 MYA (Supplementary

Fig. 6). Therefore, as seen in the age analysis of the TEs, and now the pair wise analysis of syntenic TEs, there was a recent burst of TEs in the cannabis genome.

The 1:1 syntenic depth suggests that either cannabis has not had a WGD event recently, or it has been aggressively fractionated back to diploid state with few duplicated regions. Alignment with amborella, which is the sister of the eudicots and lacks a WGD [135], resulted in a 3:1 syntenic depth consistent with cannabis having a WGD event after the split with amborella. It has been shown that grape (*Vitis vinifera*) has only the lambda WGT (hexaploidization) that is shared by many eudicots [136]. Cannabis has a 1:1 syntenic depth with grape consistent with it sharing the WGT with grape but not having another polyploidy event. A high quality genome is also available for the sister species hop (*Humulus lupulus*) [137]. We found that hop and cannabis share a similar 1:1 syntenic depth as we saw with grape, suggesting that both cannabis and hop share the lambda WGT, but do not have any subsequent WGD events (Supplementary Fig. 6).

When the TEs were removed from the cannabis self-self syntenic pairs, the off-diagonal (WGD remnant pairs) were consistent with the cannabis genome experiencing a polyploidization event around 100 MYA, which coincides with the lambda WGT shared with grape and hop (Supplementary Fig. 6). The self-self syntenic pairs for grape and amborella were consistent with them separating around 100 and 150 MYA respectively, further supporting the alignment to the cannabis genome (Supplementary Fig. 6). Since hop and cannabis share the lambda WGT but do not have any other polyploidization events, the hop self-self syntenic pairs should also be around 100 MYA apart. However, what we observe is similar to cannabis with two peaks around 10 and 30 MYA (Supplementary Fig. 6), suggesting the hop genome also has a large number of TEs that are not masked. Taken together these results showed that cannabis has one WGT event shared with grape and hop, as well as many other eudicots, but neither cannabis nor hop have had a more recent polyploidization event. However, both the cannabis and hop genomes have been shaped by recent bursts of TEs that may have played a role in separating these species.

The genes that were retained in the cannabis genome after the lambda WGT and fractionation were analyzed for gene ontology (GO) enrichment to identify potential pathways evolutionarily conserved. There were roughly 9,000 genes retained in the cannabis genome and these genes resulted in >700 overrepresented GO terms with the most significant terms relating to disease resistance and response to the environment (Supplementary Fig. 6). While the cannabis genome does not have a recent WGD, it does have a number of tandem duplicated (TD) genes that also play a role in the recent ks peaks. GO overrepresentation analysis of the TD genes revealed that much like the retained genes, disease resistance, response to the environment as well as secondary metabolism were enriched, which suggests a role for these genes in adaptive responses (Supplementary Fig. 6).

Supplementary References

1. Simão, F. A., Waterhouse, R. M., Ioannidis, P., Kriventseva, E. V. & Zdobnov, E. M. BUSCO: assessing genome assembly and annotation completeness with single-copy orthologs. *Bioinformatics* **31**, 3210–3212 (2015).
2. Divashuk, M. G., Alexandrov, O. S., Razumova, O. V., Kirov, I. V. & Karlov, G. I. Molecular Cytogenetic Characterization of the Dioecious Cannabis sativa with an XY Chromosome Sex Determination System. *PLoS ONE* vol. 9 e85118 Preprint at https://doi.org/10.1371/journal.pone.0085118 (2014).
3. Colt, K. *et al.* Telomere Length in Plants Estimated with Long Read Sequencing. *bioRxiv* 2024.03.27.586973 (2024) doi:10.1101/2024.03.27.586973.
4. Ren, G. *et al.* Large-scale whole-genome resequencing unravels the domestication history of Cannabis sativa. *Sci Adv* **7**, (2021).
5. Li, H. Minimap2: pairwise alignment for nucleotide sequences. *Bioinformatics* **34**, 3094–3100 (2018).
6. Goel, M., Sun, H., Jiao, W.-B. & Schneeberger, K. SyRI: finding genomic rearrangements and local sequence differences from whole-genome assemblies. *Genome Biol.* **20**, 277 (2019).
7. Goel, M. & Schneeberger, K. plotsr: visualizing structural similarities and rearrangements between multiple genomes. *Bioinformatics* **38**, 2922–2926 (2022).
8. Woods, P., Price, N., Matthews, P. & McKay, J. K. Genome-wide polymorphism and genic selection in feral and domesticated lineages of Cannabis sativa. *G3* **13**, (2022).
9. van Velzen, R. & Schranz, M. E. Origin and Evolution of the Cannabinoid Oxidocyclase Gene Family. *Genome Biol. Evol.* **13**, (2021).
10. Laverty, K. U. *et al.* A physical and genetic map of Cannabis sativa identifies extensive rearrangements at the THC/CBD acid synthase loci. *Genome Res.* **29**, 146–156 (2019).
11. Smith, C. J., Vergara, D., Keegan, B. & Jikomes, N. The phytochemical diversity of

commercial Cannabis in the United States. *PLoS One* **17**, e0267498 (2022).

12. Barro-Trastoy, D. & Köhler, C. Helitrons: genomic parasites that generate developmental novelties. *Trends Genet.* (2024) doi:10.1016/j.tig.2024.02.002.
13. Grassa, C. J. *et al.* A new Cannabis genome assembly associates elevated cannabidiol (CBD) with hemp introgressed into marijuana. *New Phytol.* (2021) doi:10.1111/nph.17243.
14. Gabriel, L., Hoff, K. J., Brůna, T., Borodovsky, M. & Stanke, M. TSEBRA: transcript selector for BRAKER. *BMC Bioinformatics* **22**, 566 (2021).
15. Cantalapiedra, C. P., Hernández-Plaza, A., Letunic, I., Bork, P. & Huerta-Cepas, J. eggNOG-mapper v2: Functional Annotation, Orthology Assignments, and Domain Prediction at the Metagenomic Scale. *Mol. Biol. Evol.* **38**, 5825–5829 (2021).
16. Gershenzon, J. & Dudareva, N. The function of terpene natural products in the natural world. *Nat. Chem. Biol.* **3**, 408–414 (2007).
17. LaVigne, J. E., Hecksel, R., Keresztes, A. & Streicher, J. M. Cannabis sativa terpenes are cannabimimetic and selectively enhance cannabinoid activity. *Sci. Rep.* **11**, 8232 (2021).
18. Zager, J. J., Lange, I., Srividya, N., Smith, A. & Lange, B. M. Gene Networks Underlying Cannabinoid and Terpenoid Accumulation in Cannabis. *Plant Physiol.* **180**, 1877–1897 (2019).
19. Watts, S. *et al.* Cannabis labelling is associated with genetic variation in terpene synthase genes. *Nat Plants* **7**, 1330–1334 (2021).
20. Allen, K. D. *et al.* Genomic characterization of the complete terpene synthase gene family from Cannabis sativa. *PLoS One* **14**, e0222363 (2019).
21. Hazekamp, A., Tejkalová, K. & Papadimitriou, S. Cannabis: From Cultivar to Chemovar II—A Metabolomics Approach to Cannabis Classification. *Cannabis and Cannabinoid Research* **1**, 202–215 (2016).
22. Booth, J. K., Page, J. E. & Bohlmann, J. Terpene synthases from Cannabis sativa. *PLoS One* **12**, e0173911 (2017).

23. Booth, J. K. *et al.* Terpene Synthases and Terpene Variation in Cannabis sativa. *Plant Physiol.* **184**, 130–147 (2020).
24. Osuna-Cruz, C. M. *et al.* PRGdb 3.0: a comprehensive platform for prediction and analysis of plant disease resistance genes. *Nucleic Acids Res.* **46**, D1197–D1201 (2018).
25. Pépin, N., Hebert, F. O. & Joly, D. L. Genome-Wide Characterization of the MLO Gene Family in Cannabis sativa Reveals Two Genes as Strong Candidates for Powdery Mildew Susceptibility. *Front. Plant Sci.* **12**, 729261 (2021).
26. Sirangelo, T. M., Ludlow, R. A. & Spadafora, N. D. Molecular Mechanisms Underlying Potential Pathogen Resistance in Cannabis sativa. *Plants* **12**, (2023).
27. Garfinkel, A. R. *et al.* Genetic Mapping of SNP Markers and Candidate Genes Associated with Day-Neutral Flowering in Cannabis sativa L. *bioRxiv* 2023.04.17.537043 (2023) doi:10.1101/2023.04.17.537043.
28. Beutler, J. A. & Marderosian, A. H. Chemotaxonomy of Cannabis I. Crossbreeding between Cannabis sativa and C. ruderalis, with analysis of cannabinoid content. *Econ. Bot.* **32**, 387–394 (1978).
29. Coyne, J. A., Coyne, H. A. & Allen Orr, H. *Speciation*. (Oxford University Press, Incorporated, 2004).
30. Salomé, P. A. *et al.* The recombination landscape in Arabidopsis thaliana F2 populations. *Heredity* **108**, 447–455 (2012).
31. Nadir, S. *et al.* An overview on reproductive isolation in Oryza sativa complex. *AoB Plants* **10**, ly060 (2018).
32. Toth, J. A., Stack, G. M., Carlson, C. H. & Smart, L. B. Identification and mapping of major-effect flowering time loci Autoflower1 and Early1 in Cannabis sativa L. *Front. Plant Sci.* **13**, 991680 (2022).
