# Supplementary material for: Domesticated cannabinoid synthases amid a wild mosaic cannabis pangenome
Source: Nature. 2025 May 28;643(8073):1001–10. doi: 10.1038/s41586-025-09065-0 (PMC12286863; doi:10.1038/s41586-025-09065-0)
Supplement: Supplementary file 2 — Reporting Summary [file 41586_2025_9065_MOESM2_ESM.pdf]

nature portfolio

Corresponding author(s): Ryan Lynch, Todd Michael, Lillian Padgit-Cobb

Last updated by author(s): 2025-4-14

Reporting Summary

Nature Portfolio wishes to improve the reproducibility of the work that we publish. This form provides structure for consistency and transparency in reporting. For further information on Nature Portfolio policies, see our Editorial Policies and the Editorial Policy Checklist.

Statistics

For all statistical analyses, confirm that the following items are present in the figure legend, table legend, main text, or Methods section.

n/a	Confirmed	
☐	☒	The exact sample size (*n*) for each experimental group/condition, given as a discrete number and unit of measurement
☐	☒	A statement on whether measurements were taken from distinct samples or whether the same sample was measured repeatedly
☐	☒	The statistical test(s) used AND whether they are one- or two-sided<br>*Only common tests should be described solely by name; describe more complex techniques in the Methods section.*
☐	☒	A description of all covariates tested
☐	☒	A description of any assumptions or corrections, such as tests of normality and adjustment for multiple comparisons
☐	☒	A full description of the statistical parameters including central tendency (e.g. means) or other basic estimates (e.g. regression coefficient) AND variation (e.g. standard deviation) or associated estimates of uncertainty (e.g. confidence intervals)
☐	☒	For null hypothesis testing, the test statistic (e.g. *F*, *t*, *r*) with confidence intervals, effect sizes, degrees of freedom and *P* value noted<br>*Give P values as exact values whenever suitable.*
☒	☐	For Bayesian analysis, information on the choice of priors and Markov chain Monte Carlo settings
☐	☒	For hierarchical and complex designs, identification of the appropriate level for tests and full reporting of outcomes
☐	☒	Estimates of effect sizes (e.g. Cohen's *d*, Pearson's *r*), indicating how they were calculated

Our web collection on statistics for biologists contains articles on many of the points above.

Software and code

Policy information about availability of computer code

Data collection	Sequencing data were recorded as fastq files (see Methods), quantitative phenotypic data was collected manually in fields and greenhouses and recorded in Microsoft Excel (v18.85) (see Methods).
Data analysis	Each analysis in our study uses software that is described and referenced in our Methods section. Version information, parameters, and settings are also provided to allow for a clear understanding of how each analysis was performed. Scripts and analysis pipelines are available at https://github.com/anthony-aylward/CannabisPangenomeShared and https://github.com/padgittl/CannabisPangenomeAnalyses.<br><br>3D-DNA pipeline (version 180922), Apptainer (v1.1.8), assembly-stats (v1.0.1), bedops , bedtools , Bcftools , Blastn , Braker (v2.1.6), BUSCO (v5.4.3), Cactus (v2.6.7), CD-HIT , diamond (v2.1.4), Dorado (v0.3.4), EDTA (v1.9.6), eggNOG-mapper (v2.0.1), FALCON Unzip (PacBio SMRT Tools 9.0), FastTree , fastp , Freebayes , GAPIT (v3), Geneious Basic , genespace (v0.9.3), Hifiasm (v0.16.162), HiCanu (v2.2), HISAT2 (v2.2.1), IQ-TREE (v1.6.12), Juicebox (v1.11.086), Juicer (v1.6.2), MAFFT (v7.505), MEGA (v11), Minimap , Minimap2 (v2.24), Minigraph-Cactus , modkit , Nextflow (v24.04.3.5916), nf-core/pangenome (v1.1.2), OrthoFinder (v2.5.4), PanKmer , PGGB , plink , plotsr , R (v4.2.2), RagTag (v2.1.0), Salmon (v1.6.0), samtools , snakemake , Sourmash (v4.6.182), syRI , Tandem Repeat Finder (v4.09), TopGO , TranslatorX , Trim Galore , TSEBRA , vcfbub , vcftools , vg (v1.61.0)

For manuscripts utilizing custom algorithms or software that are central to the research but not yet described in published literature, software must be made available to editors and reviewers. We strongly encourage code deposition in a community repository (e.g. GitHub). See the Nature Portfolio guidelines for submitting code & software for further information.

Data

Policy information about availability of data

All manuscripts must include a data availability statement. This statement should provide the following information, where applicable:

- Accession codes, unique identifiers, or web links for publicly available datasets
- A description of any restrictions on data availability
- For clinical datasets or third party data, please ensure that the statement adheres to our policy

Data Availability
The NCBI BioProject ID for the cannabis pangenome is PRJNA1140642. All of the pangenome sequencing data at NCBI SRA is under the BioProject accession PRJNA904266. The BioProject accession IDs for EH23a and EH23b are PRJNA1111955 and PRJNA1111956, respectively. Genomes and annotation files for all 193 assemblies, including links to corresponding U.S. National Plant Germplasm System accessions are available from: resources.michael.salk.edu. Orthobrowser and Genome Jbrowse instances are hosted at: resources.michael.salk.edu. Input and output files for graph pangenomes are available at resources.michael.salk.edu. Annotations for R-genes, terpene synthases, cannabinoid synthases, and additional genome visualizations are available from: figshare.com/projects/Cannabis_Pangenome/205555 and https://doi.org/10.25452/figshare.plus.c.7248427.v1. Links to specific genome datasets are provided in Supplementary Table 1: https://doi.org/10.6084/m9.figshare.25869319.v1.

Research involving human participants, their data, or biological material

Policy information about studies with human participants or human data. See also policy information about sex, gender (identity/presentation), and sexual orientation and race, ethnicity and racism.

Reporting on sex and gender	NA
Reporting on race, ethnicity, or other socially relevant groupings	NA
Population characteristics	NA
Recruitment	NA
Ethics oversight	NA

Note that full information on the approval of the study protocol must also be provided in the manuscript.

Field-specific reporting

Please select the one below that is the best fit for your research. If you are not sure, read the appropriate sections before making your selection.

☐ Life sciences ☐ Behavioural & social sciences ☒ Ecological, evolutionary & environmental sciences

For a reference copy of the document with all sections, see nature.com/documents/nr-reporting-summary-flat.pdf

Ecological, evolutionary & environmental sciences study design

All studies must disclose on these points even when the disclosure is negative.

Study description	Cannabis sativa plants were cultivated and crossed in controlled enviroments and fields in, OR, CO and CA.
Research sample	Cannabis sativa pangenome samples were selected from multiple sources to maximize the genetic diversity, history and agronomic value. A large portion of the pangenome comes from the Oregon CBD (OCBD) breeding program that includes elite cultivars; foundational marjuana lines potentially originating from the 1970s, 80s, 90s to present; and elite trios used for different aspects of the breeding program. The remaining cultivars come from the United States Department of Agriculture (USDA) Germplasm Resource Information Network (GRIN) and German federal genebank (IPK Gatersleben) repositories, as well as collections made by the Salk Institute from various breeders. The pangenome includes European and Asian fiber and seed hemp, feral populations, North American marijuana (type I), and North American high cannabinoid yielding (CBD or CBG) hemp (type III and IV). Additional cannabinoid diversity is represented with chemotypes presenting high expression of pentyl or propyl (varin) homologs of CBD or THC, and cannabinoid free (type V) plants. Flowering time variation is also captured with the inclusion of both regular short day and day neutral (autoflowering) phenotypes (Supplemental Table 1).
Sampling strategy	In addition to the whole genome sequencing data described above, ERBxHO40_23 was self-pollinated using STS induced masculinization of select flowers, to create an F2 mapping population. From this F2 population, individuals were scored for autoflower, varin content, and sequenced using Illumina 100 bp reads by NRGene (Nrgene Technologies Ltd, Israel). Illumina WGS

	genotyping runs were performed on 288 plants from this population, plus the ERBxHO40_23 parent. Sample size for this population was dictated by available greenhouse space and assessment of prior mapping studies.
Data collection	For HMW DNA extractions leaf material was sampled as described in the methods. For RNAseq libraries samples were collected from six tissues during the course of of the ERBxHO40_23 individual: development: shoot tips, roots, late flower, leaf under short day, leaf under long day, and early flower
Timing and spatial scale	For RNAseq libraries, late flower = 8 weeks under 8-hour light / 16-hour dark conditions, leaf under short day = 4 weeks under 8-hour light and 16-hour dark, leaf under long day = 4 weeks under 12 hour light / 12 hour dark, and early flower 2 weeks under 8-hour light / 16-hour dark conditions.
Data exclusions	NA
Reproducibility	NA
Randomization	NA
Blinding	NA

Did the study involve field work? ☐ Yes ☒ No

Reporting for specific materials, systems and methods

We require information from authors about some types of materials, experimental systems and methods used in many studies. Here, indicate whether each material, system or method listed is relevant to your study. If you are not sure if a list item applies to your research, read the appropriate section before selecting a response.

Materials & experimental systems

n/a	Involved in the study	
☒	☐	Antibodies
☒	☐	Eukaryotic cell lines
☒	☐	Palaeontology and archaeology
☒	☐	Animals and other organisms
☒	☐	Clinical data
☒	☐	Dual use research of concern
☐	☒	Plants

Methods

n/a	Involved in the study	
☒	☐	ChIP-seq
☒	☐	Flow cytometry
☒	☐	MRI-based neuroimaging

Plants

Seed stocks	Links to corresponding U.S. National Plant Germplasm System accessions are available from: resources.michael.salk.edu
Novel plant genotypes	Breeding techniques are described clearly in the methods and a pedigree diagram is provided in Supplementary Figure 1.
Authentication	NA
